# Integrin beta8 (ITGB8) activates VAV-RAC1 signaling via FAK in the acquisition of endometrial epithelial cell receptivity for blastocyst implantation

**DOI:** 10.1038/s41598-017-01764-7

**Published:** 2017-05-15

**Authors:** Vijay Kumar, Upendra Kumar Soni, Vineet Kumar Maurya, Kiran Singh, Rajesh Kumar Jha

**Affiliations:** 1 Division of Endocrinology, CSIR-Central Drug Research Institute, Sector-10, Jankipuram Extension, Sitapur Road, Lucknow, 226031 U.P. India; 2 Department of Molecular & Human Genetics, Banaras Hindu University (BHU), Varanasi, UP India

## Abstract

Integrin beta8 (ITGB8) is involved in the endometrial receptivity. The blastocyst first interacts with the luminal endometrial epithelial cells during its implantation; therefore, we have investigated the signaling of ITGB8 via FAK and VAV-RAC1 in the endometrial epithelial cells. Integrin beta8 was found elevated in epithelial cells at late-pre-receptive (day4, 1600 h) and receptive (day5, 0500 h) stages of endometrial receptivity period in the mouse. Integrins downstream molecule FAK has demonstrated an increased expression and phosphorylation (Y397) in the endometrium as well as in the isolated endometrial epithelial cells during receptive and post-receptive stages. Integrin beta8 can functionally interact with FAK, VAV and RAC1 as the levels of phosphorylated-FAK, and VAV along with the RAC-GTP form was reduced after ITGB8 knockdown in the endometrial epithelial cells and uterus. Further, VAV and RAC1 were seen poorly active in the absence of FAK activity, suggesting a crosstalk of ITGB8 and FAK for VAV and RAC1 activation in the endometrial epithelial cells. Silencing of *ITGB8* expression and inhibition of FAK activity in the Ishikawa cells rendered poor attachment of JAr spheroids. In conclusion, ITGB8 activates VAV-RAC1 signaling axis via FAK to facilitate the endometrial epithelial cell receptivity for the attachment of blastocyst.

## Introduction

Endometrial receptivity is a predefined and restricted period known as the ‘window of endometrial receptivity’ which is crucial to facilitate the blastocyst implantation and induces various mechanisms originating from the blastocyst and endometrium. This is a complex process to bring an intimate crosstalk between activated/implanting/competent blastocyst and a receptive uterus or endometrium. A synchrony between the competent blastocyst and a receptive endometrium is induced to achieve an optimal blastocyst implantation^1–3^ in consequence the pregnancy is established.

Integrins have been known as the adhesion molecules that mediate the blastocyst attachment and downstream signaling activation in the uterus. Integrin alpha v beta3 is expressed in the uterus during its receptivity stages^4, 5^. Integrins are well documented heterodimeric transmembrane receptor proteins that link the extracellular matrix (ECM) to the cytoskeleton to regulate the cell shape, migration, and survival. Binding of the integrins to ECM ligands trigger the formation of focal adhesions (FAs), multi-protein signaling complexes that bridge the integrin cytoplasmic tails with the actin cytoskeleton^6^. Integrin beta (ITGB) family member beta8 has been reported in the epithelial cell growth regulation^7–9^ and our recent report has documented its role in the endometrial receptivity for embryo implantation process^10^, but we could not establish any detail downstream signaling in particular to the endometrial epithelial cells.

Although integrins can serve as extracellular matrix (ECM) receptor, it can also trigger downstream molecules like focal adhesion kinase (FAK) and propagate the signaling cascade. Focal adhesion kinase (FAK) is a 125 kDa non-receptor tyrosine kinase, which acts as a scaffold at sites of cell attachment to the extracellular matrix (ECM) and is activated following binding of integrins to ECM or upon growth factor stimulation including that mediated by VEGF^8, 11, 12^. FAK is an important modulator of angiogenesis as the study of transgenic mouse models indicated that both the expression and activity of FAK are essential in the endothelial cells for the formation of new blood vessel network during embryonic development^13–15^. It is well studied key component of the signal transduction pathway, which is triggered/activated by the integrins. Aggregation of FAK with integrins and ECM/cytoskeleton proteins at focal contacts is responsible for FAK activation and its auto-phosphorylation at cytoplasmic tails by integrins in cell adhesion event^16, 17^.

The activity of FAK is found to be associated with VAV2-mediated RAC1 activation^18^ and RAC1 has been demonstrated in the decidualization associated signaling^19, 20^. FAK is distributed differentially on endometrial cells during the process of embryo attachment^21^ and is expressed during decidualization^22^ and blastocyst outgrowth predominantly^23^. Therefore, it acts as a potential biochemical determinant of trophoblast invasion^24^. Its expression during the human menstruation cycle has already been reported^25^. A study by Hanashi *et al*., showed the role of FAK in the decidua under *in vitro* conditions^26^, but fails to provide a detailed picture. Importantly, the endometrial luminal epithelial cells sense the implanted blastocyst and accommodate it for pregnancy establishment^27, 28^ and ITGB3 has been vital in this process^29, 30^. Further, recently one of our study has demonstrated a prominent expression of ITGB8 in the endometrial epithelial cells^10^. However, apart from the adhesion process of integrin during the lodging process of a blastocyst on the endometrial cells to facilitate the implantation process, they also may trigger the intracellular signaling pathways *via* various biochemical messengers, but this needs further investigation, which is being reported in the present study.

Herein, we report the FAK-VAV-RAC1 signaling axis operation in the endometrial epithelial cells in response to the ITGB8 signaling during acquisition of endometrial epithelial cell receptivity for the establishment of embryo implantation.

## Results

### Integrin beta8 is upregulated during the receptive stage in the uterine epithelial cells during window of endometrial receptivity period in a mouse model and directs its downstream signaling through Focal Adhesion Kinase (FAK)

**Figure 1:**
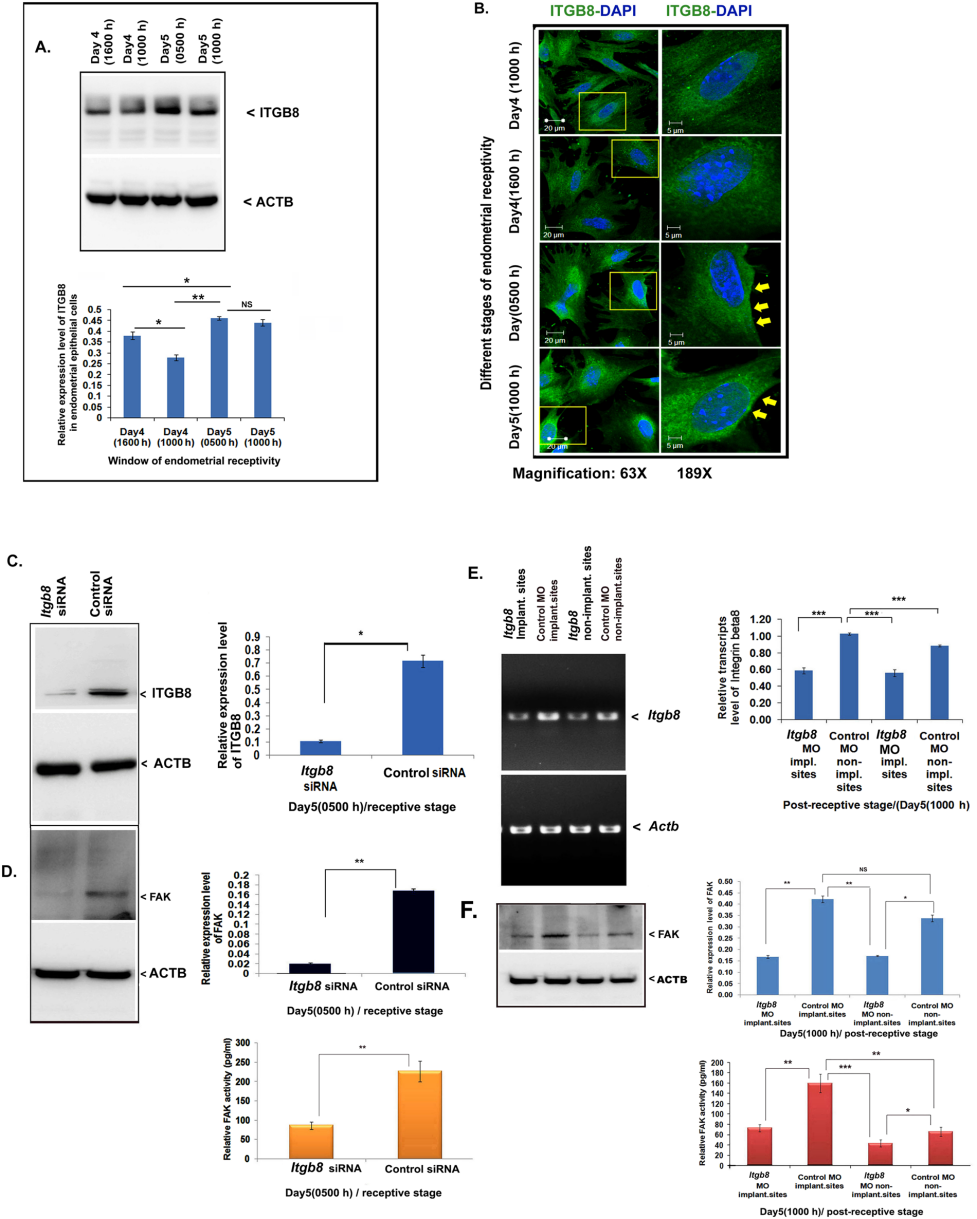
Integrin beta8 regulates the activity of FAK in the endometrial epithelial cells during their receptivity. (**A**) Integrin beta8 expression analysis was performed on the endometrial epithelial cells with the help of immunoblotting and densitometry. (**B**) Employing immunofluroescence, the localization of integrin beta8 was performed through confocal laser scanning microscope in the endometrial epithelial cells of mouse origin. (**C**) Silencing of *Itgb8* from endometrial epithelial cells was confirmed by immunoblotting and densitometry. (**D**) The expression level of phosphorylated and total forms of FAK was examined in response to *Itgb8* silencing in the endometrial epithelial cells using immunoblotting and densitometry. (**E**,**F**) Similarly, FAK activity was determined by way of phosphorylation (Y-397) level determination by ELISA in the endometrial tissue protein extract from day5, 1000 h stage post-*Itgb8* silencing at day4, 1000 h. Actin beta was used as the loading control to normalize the western blot values. (**p < 0.01,*p < 0.05, NS p > 0.05). Magnification of the image was at 63X and 189X. Scale bar; 20 and 5 µm respectively.

In our recent report, we have demonstrated the expression of ITGB8 in the endometrium and it was predominant in the luminal epithelial cells, which is essential for embryo implantation process^10^. Importantly, ITGB8 controls the TGF-B activation, which is also one of the crucial signaling in the acqusition of endometrial receptivity for blastocyst implantaiton^10, 31^. However, the downstream signaling triggered by the ITGB8 in the receptive endometrial epithelial cells is still unknown. Therefore, we aimed to analyze the same using mouse model. We observed the presence of ITGB8 in the endometrial epithelial cells throughout the stages of early pregnancy (window of endometrial receptivity) (Fig. 1A). Endometrial epithelial cells from pre-implantation stage showed a basal level of ITGB8 (Fig. 1A) followed by increased expression of ITGB8 at receptive (early and late) stages (p < 0.005). At the advance stage (post-receptive), the expression level of ITGB8 exhibited a declined trend (Fig. 1A).

Our result exhibited the localization of ITGB8 throughout the endometrial receptivity period and showed the presence ubiquitously in the cells (Fig. 1B). The fluorescence signal of integrin beta8 seems to be concentrated in vesicular compartments (Fig. 1B).

In our earlier report, we have found ITGB8 mediated regulation of STAT3 and ITGB3 expression in the mouse endometrium^10^. Based on the above leads we further planned to analyze the integrin beta8 dependent regulation of STAT3 and ITGB3 signaling in the isolated endometrial epithelial cells as these cells serve as one of the first interaction sites for the blastocyst attachment. To uncover the mechanism, we knocked down the ITGB8 from the epithelial cells (Fig. 1C) and confirmed by the immunoblotting. Herein, we observed that *Itgb*8 knock-down led (Fig. 1C) to a reduced expression of total and phosphorylated forms of STAT3 (activity) (Fig. S1A). Downregulation of ITGB3 in the *Itgb*8 depleted endometrial epithelial cells was also observed (Fig. S1B).

Further, to elucidate the functional interaction of ITGB8 with FAK, we silenced the expression of *Itgb*8 in the endometrial epithelial cells (Fig. 1C); in consequence, the expression of total and phosphorylated forms of FAK was found to be decreased significantly (Fig. 1D).

Later, we extended our observations under *in vivo* conditions using mouse model. We first knockdown the *Itgb8* at the day4, 1000 h (p.c.) and found the same phenotype (Fig. S1C) as seen in our previous study^10^. The implantation sites were reduced along with the numbers of the blastocyst (Fig. S1C). Post-*Itgb8* knockdown by morpholino oligo at day5 (1000 h), we found suppressed levels of the transcript of the *Itgb8* in the implantation and non-implantation sites (Fig. 1E) than those of scrambled or control MO, which confirmed the knockdown of *Itgb8* at day5, 1000 h.

FAK expression was decreased post-*Itgb8* depletion in the uterus at day5, 1000 h stage (Fig. 1F). Further, we assayed the activity of FAK by way of phosphorylated form level determination and interestingly, we found the compromised activity of FAK in response to *Itgb8* depletion (Fig. 1F).

### Focal Adhesion Kinase (FAK) is associated with endometrial epithelial cells preparation for the receptivity establishment

FAK is one of the important players during embryo invasion and expressed in the uterine luminal, glandular epithelial cells^32^. Focal adhesion kinase (FAK) is a central protein tyrosine kinase (PTK) involved in integrin signaling^33^ and during its activation, FAK gets phosphorylated at Y397 residue. There are reports to show the interaction of FAK and integrins, but not in the context of ITGB8^6^ and our results confirmed the interaction of ITGB8 and FAK as silencing of *Itgb*8 exhibited poor expression and activity of FAK in the endometrial cells (Fig. 1F).

**Figure 2:**
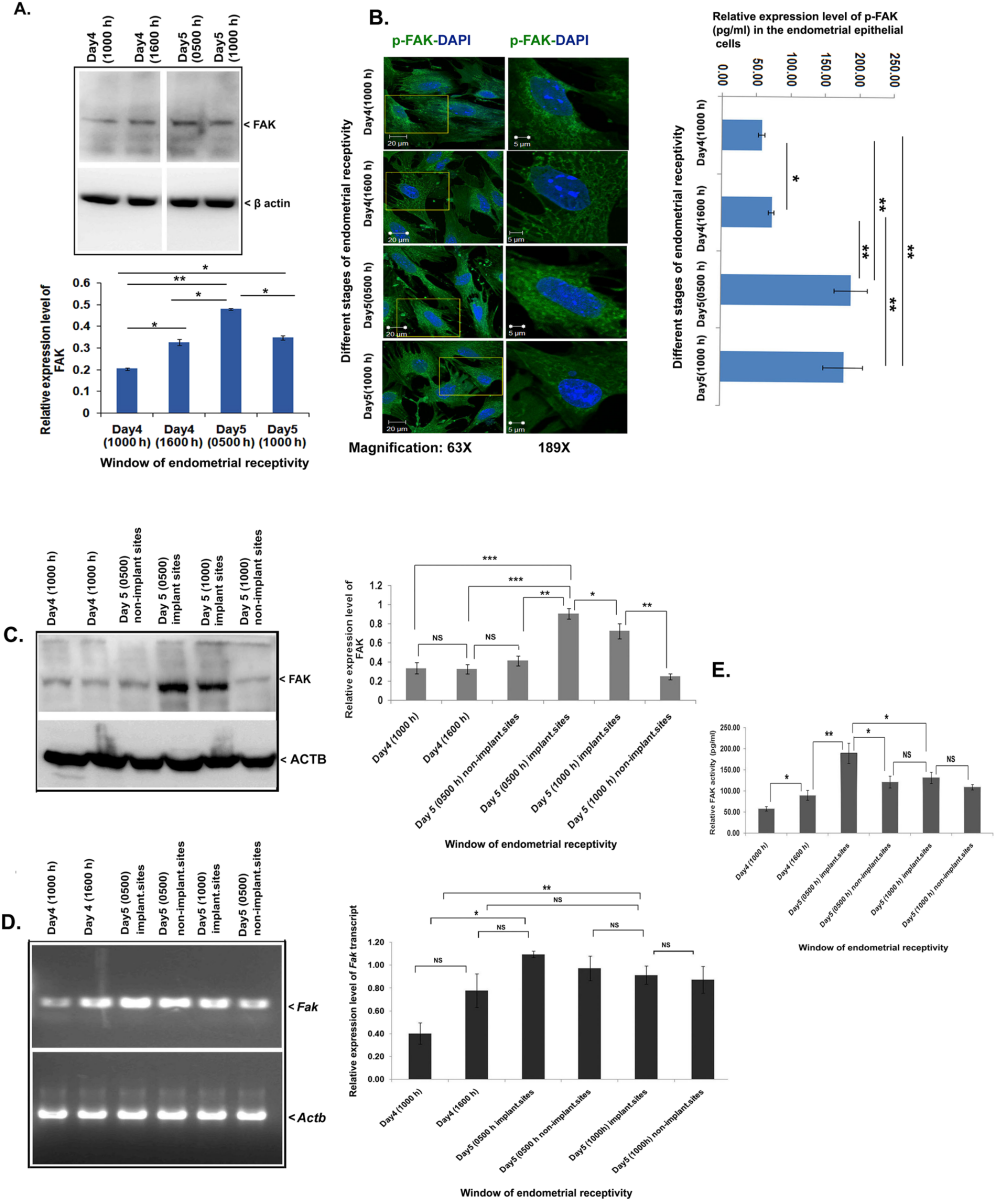
FAK expression and activity was upregulated in the endometrium during the phases of receptivity for embryo implantation. (**A**) FAK is analyzed by immunoblotting and densitometric analysis in the endometrial epithelial cells. (**B**) Later, we immunolocalized the phosphorylated-FAK in the epithelial cells and performed ELISA for phosphorylated-FAK (active) levels analysis in the cells. (**C**) FAK expression was studied using immunoblotting and densitometric analysis in the endometrium during the endometrial (Uterine) receptivity phases. (**D**) The transcript level of *Fak* was also examined in the endometrium with the help of PCR and densitometric analysis during endometrial receptivity phases. (**E**) During endometrial receptivity phases, using ELISA based method, the FAK activity assay was done by determining the phosphorylation (Y/Tyr397) level. (***p < 0.001, **p < 0.01,*p < 0.05, NS p > 0.05).

Next, we analyzed the expression level of FAK in the uterus by immunoblotting in the uterus during different stages of endometrial receptivity as well as in the isolated endometrial epithelial cells from the receptive phase of the endometrium (Uterus). Primary (isolated and cultured for 24 hours) endometrial epithelial cells exhibited a mild increase in the expression of FAK at receptive/peri-implantation stage (Fig. 2A). Interestingly, the phosphorylated-FAK form was very strong at the receptive stage and extended to the post-receptive stage as seen through ELISA and immunolocalization analysis (Fig. 2B).

We determined the expression level of FAK in mouse uterine tissue during different stages of endometrial receptivity period. In the uterus, we observed the mRNA expression level of *Fak* in all the stages of pregnancy studied. The pre-receptive demonstrated low expression of FAK, which spiked at receptive and post-receptive stages (Fig. 2C and D). Surprisingly, the phosphorylated-FAK form was seen higher at implantation sites of the receptive stage (day5, 0500 h) than any other stage of the early or late period of endometrial receptivity (Fig. 2E).

**Figure 3:**
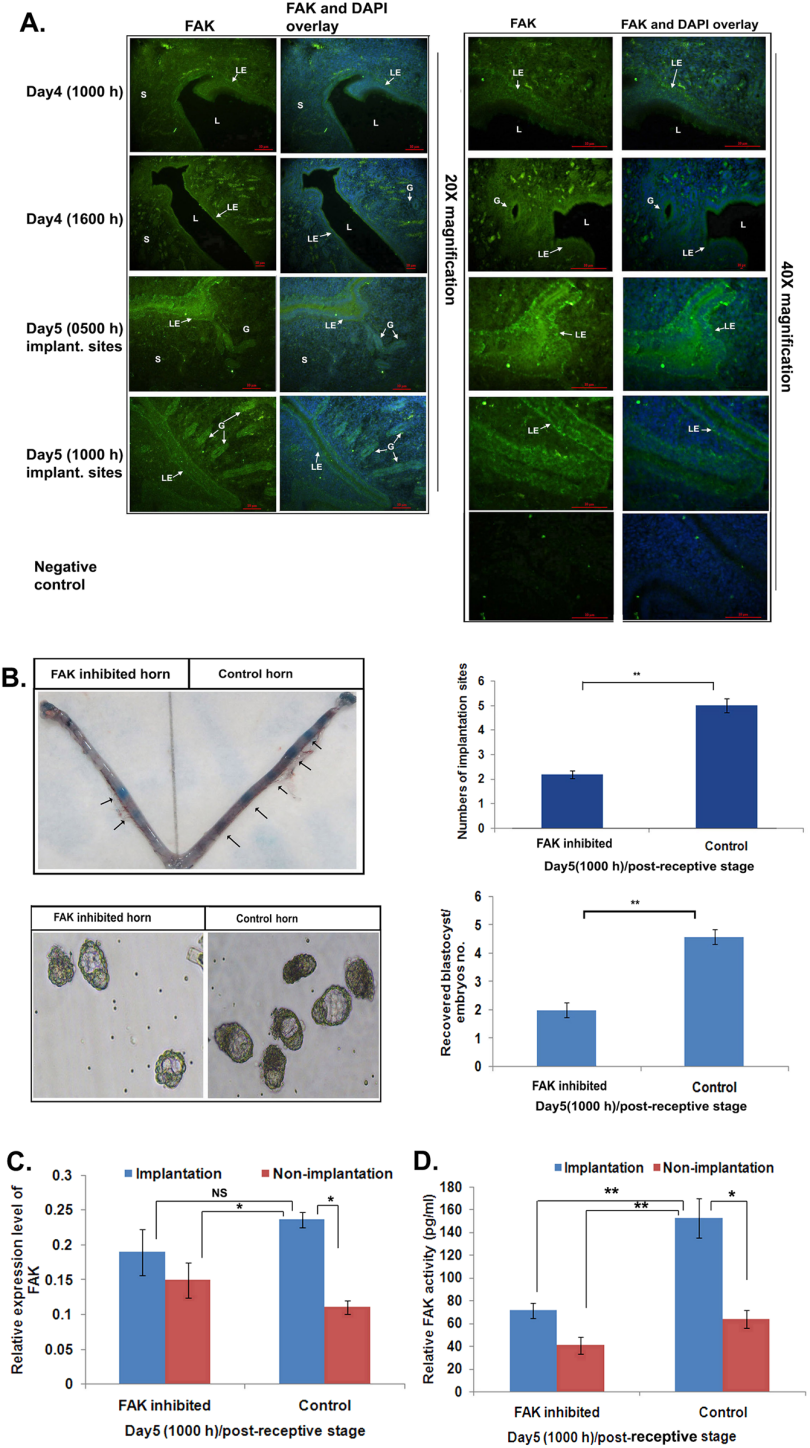
Cellular distribution of FAK was predominant in the epithelial cells in the uterus during receptivity and FAK activity inhibition can render poor implantation. (**A**) We have performed the FAK immunofluorescence in the uterus from different stages of endometrial receptivity. (**B**) FAK inhibition during the pre-receptive stage of the endometrial receptivity period exhibited poor implantation and blastocyst numbers. (**C**) The expression level of FAK was studied post-FAK activity inhibition at day 5 (1000 h) in the endometrial tissue lysate. (**D**) After the FAK activity inhibition, ELISA was performed to determine the phosphorylation level of FAK at Tyr397 during the post-implantation stage. (**p < 0.01, *p < 0.05). Magnification of objective lens was 20X and 40X with the scale bar of 10 μm.

FAK spatiotemporal distribution was seen in epithelial cells of the lumen and glandular regions (Fig. 3A) and to a detectable amount in stromal cells (Fig. 3A) in the uterus. Surprisingly, endometrial luminal epithelial cells showed demarcation of apical localization of FAK (Fig. 3A).

### Inhibition of FAK activity renders poor implantation in mouse model

To understand the functional role of FAK in receptive endometrium, we blocked the activity of FAK during pre-implantation (day4, 1000 h) using its pharmacological inhibitor, and observed the effect on day5 (1000 h)/post-receptive stage where we noticed compromised implantation sites (Fig. 3B). An expression of total FAK was unchanged in the FAK-inhibited and sham-treated implantation sites of uterine tissue (Fig. 3C); however, it was reduced in non-implantation sites of both the groups (Fig. 3C).

The phosphorylated form of FAK was decreased in the implantation as well as in the non-implantation sites of FAK-inhibitor treated groups with respect to their sham groups (Fig. 3D). Inhibition of FAK rendered the poor expression of endometrial receptivity related biochemical markers, i.e., integrin beta3 (ITGB3) (Fig. S2A) and phosphorylated-STAT3 (Fig. S2B) in the implantation regions. Furthermore, the transcript level of *Bmp2* was altered drastically in the implantation as well as in non-implantation sites of FAK activity blocked uterine tissue (Fig. S2C). Similarly, we noticed the reduced expression of total and phosphorylated forms of STAT3, and ITGB3 in the endometrial epithelial cells when FAK activity was inhibited (Fig. S2D and E).

### Integrin beta8, FAK and RAC1 are found complexed

RAC1 is a small G-protein in the RHOGTPase family that drives the conduciveness phenomenon of endometrial receptivity^20^ and inhibition of it can lead to failure of embryo implantation^34^. An earlier report has documented the activation of RAC1 by ITGB8 in the renal epithelial cells^35^.

**Figure 4:**
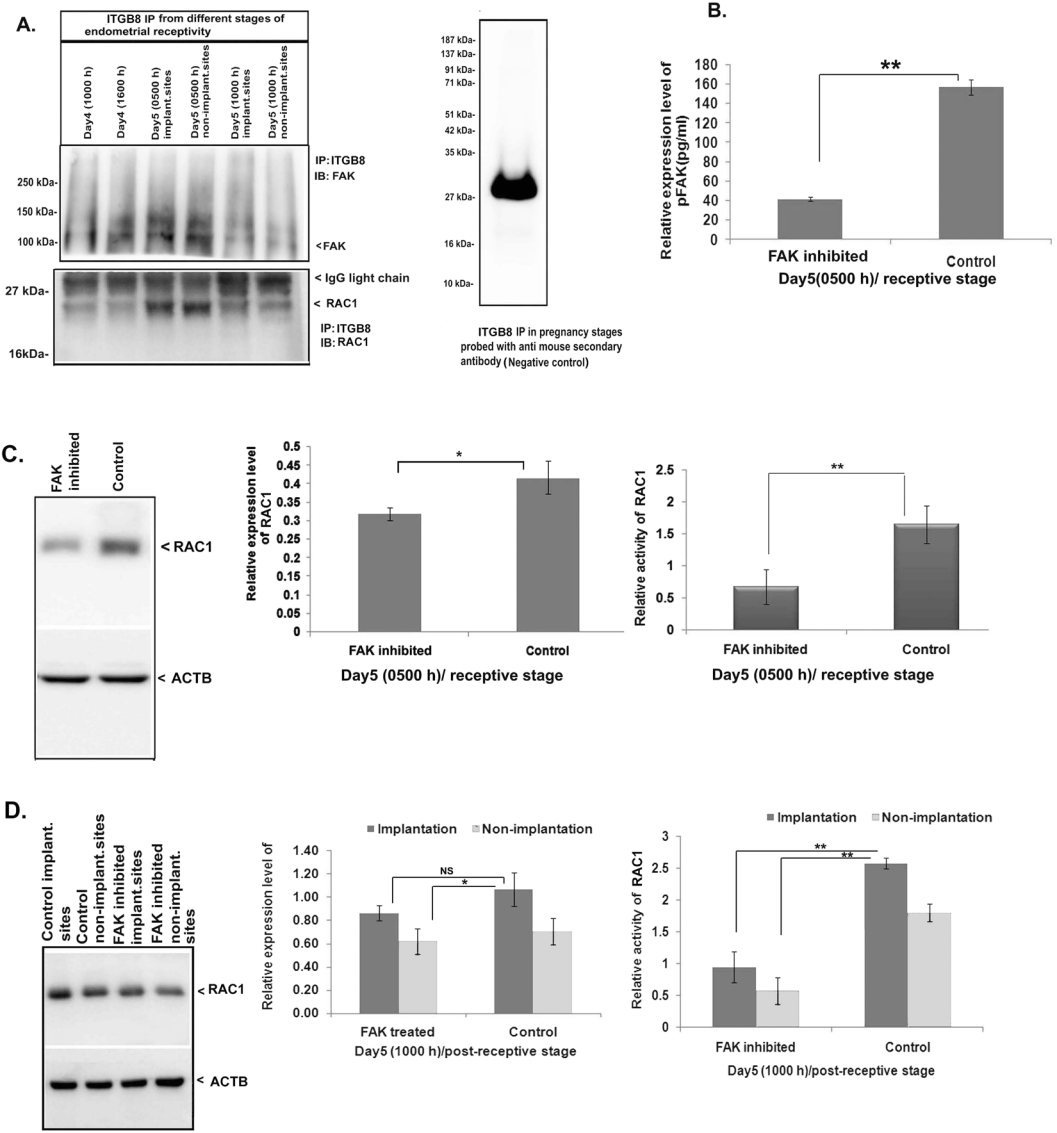
Integrin beta8 formed a complex with FAK and RAC1 in the endometrium and RAC1 is regulated by the FAK activity. (**A**) FAK was immunopositive in the immunoblot of ITGB8 IP samples from endometrial tissue. RAC1 immunoblotting was done and showed its presence on IP samples of ITGB8 from the endometrial tissue proteins. Normal mouse IgG labeling was also performed to examine any non-specific binding on the immunoblot. (**B**) Inhibition of FAK activity was performed in the endometrial epithelial cells from day5, 0500 h stage. (**C**) The expression level of RAC1 and its activity was determined in the endometrial epithelial cells from day5 (0500 h) stage in response to FAK activity inhibition. (**D**) In the endometrium, after the FAK activity inhibition, RAC1 expression and activity were analyzed in the implantation and non-implantation sites of the post-receptive stage (day5, 1000 h). (**p < 0.01,*p < 0.05, NS p > 0.05).

After, studying the functional interaction of ITGB8 with FAK, we performed an immuno-pulldown (IP) assay to know their physical interaction, if any. The immunopulldown/immunoprecipitation (IP) assay of ITGB8 is already validated in our earlier study^10^. Interestingly, we found FAK and RAC1 immunopositive bands on ITGB8 immunoprecipitated blot (Fig. 4A). Secondary antibodies (anti-rabbit IgG and mouse IgG) alone didn’t represent any protein band (Fig. 4A) as seen on immunoblots of ITGB8, FAK and RAC1. This information confirms an ITGB8 interaction with the FAK and RAC1 in the endometrial cells for the ITGB8 signaling propagation.

### GTP-bound RAC1 (active form) is also controlled by FAK in the endometrial epithelial cell during receptivity phase

Isolated and short duration cultured (24 hrs) endometrial epithelial cells from day5 (0500 h) stage showed a reduction in RAC1 activity when FAK was inhibited (Fig. 4B). Indeed, the expression of RAC1 was also mildly reduced in FAK inhibited group (Fig. 4C), but the GTP-bound form RAC1 was greatly affected (Fig. 4C). We extended our observation to FAK modulation under *in vivo* conditions and observed a decreased activity of RAC1 in the FAK inhibited implantation as well as in the non-implantation sites at day5 (1000 h) when compared with control’s implantation and non-implantation sites (Fig. 4D).

### RAC1-GTP (active form) is controlled by FAK activator, ITGB8 in the endometrial epithelial cells

**Figure 5:**
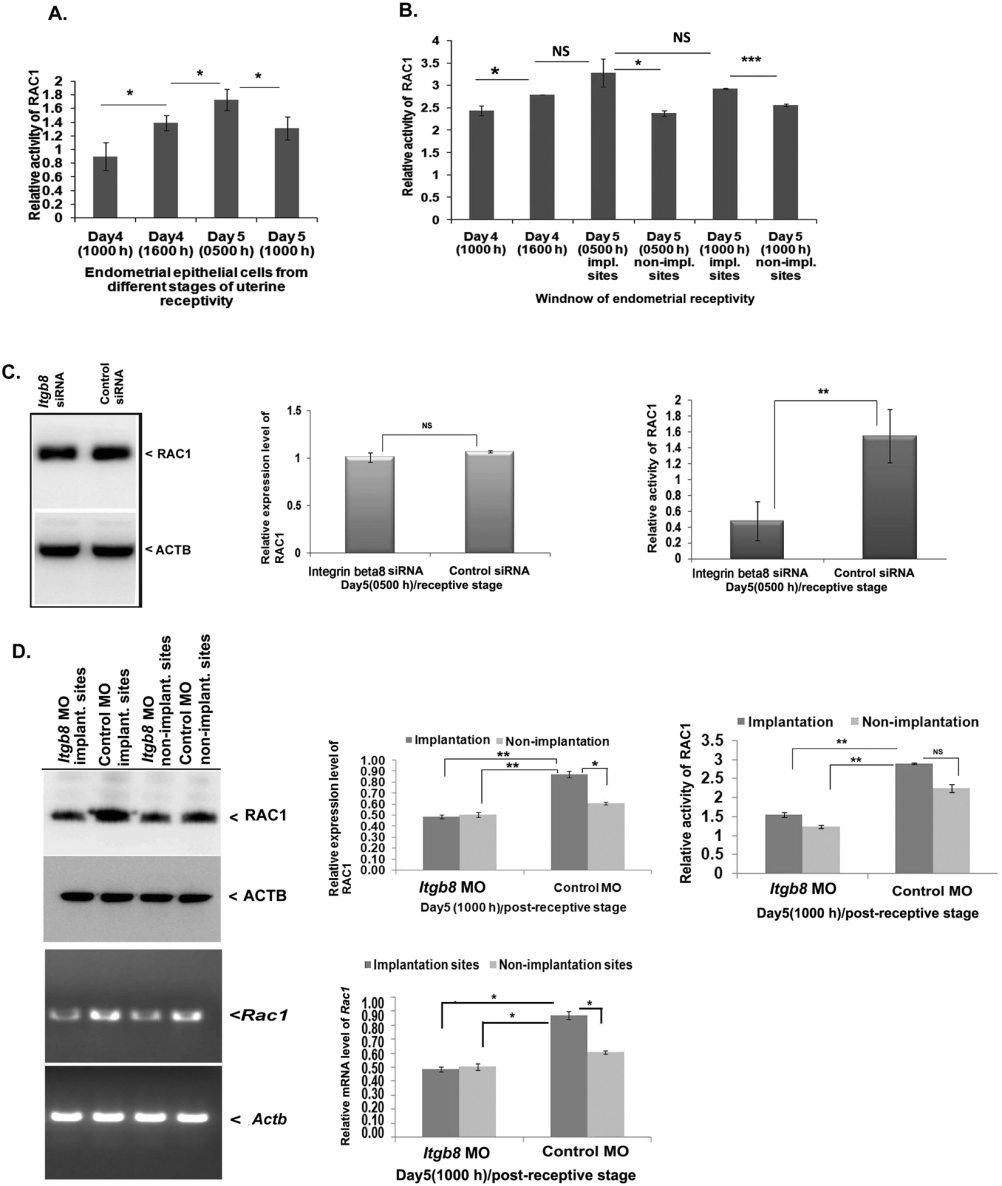
GTP-bound state of RAC1 is influenced by the ITGB8 in the endometrial epithelial cells in course of endometrial receptivity. (**A**) RAC1 activity in terms of a GTP-bound state was analyzed in the isolated and cultured endometrial epithelial cells during various stages of endometrial receptivity period. (**B**) We also determined the activity of RAC1 in the whole uterus during endometrial receptivity period. (**C**) At the same time, we assayed the expression and activity of RAC1 in the endometrial epithelial cells after *Itgb8* knockdown by its siRNA. (**D**) With the help of immunoblotting, PCR and densitometry, the expression of RAC1 and activity were determined in the whole uterus/endometrium during the post-receptivity phase in response to *Itgb8* knockdown (morpholino oligonucleotides). (***p < 0.001, **p < 0.01,*p < 0.05, NS p > 0.05).

Initially, we analyzed the activity of RAC1 by ELISA based assay in the endometrial epithelial cells during different stages of endometrial receptivity. RAC1 activity was seen elevated on day5 (0500 h) stage in endometrial epithelial cells (Fig. 5A). We also noticed that the GTP-bound form (active) of RAC1 was elevated in the implantation regions of the receptive stage uterus during blastocyst implantation period (Fig. 5B).

RAC1 activation is reported to be under the control of integrin beta8 (ITGB) signaling in the mesangial cell-myofibroblast differentiation^35^ and turned on by inactivating or dissociating RHOGDI, which is a known inhibitor of RAC1 activation^36^. Therefore, we evaluated the response of ITGB8 on RAC1 activity (GTP-bound state) in the endometrial epithelial cells and whole uterine tissue. The isolated endometrial cells from day5 (0500 h) demonstrated an approximately 3 fold reduction in the GTP-bound form (active) of RAC1 when *Itgb8* was silenced (Fig. 5C). The expression of RAC1 was also noticed to be reduced in the *Itgb8* MO group (implantation and non-implantation sites in the uterus) (Fig. 5D) than that of control group implantation sites. A similar pattern of expression was exhibited by mRNA of *Rac1* in the uterus (Fig. 5D). The GTP-bound state of RAC1 was noticed to be reduced in the *Itgb8* MO group (implantation and non-implantations sites in the uterus) in comparison to control MO groups (Fig. 5D).

### Integrin beta8 signaling regulates the expression and activation of RAC1-GEF, VAV in the endometrial epithelial cells

VAV is a guanine exchange factor for RHOGTPase and is widely reported for RAC1. The activation of VAV is noticed in the signaling of integrin, which prompted us to explore such events.

**Figure 6:**
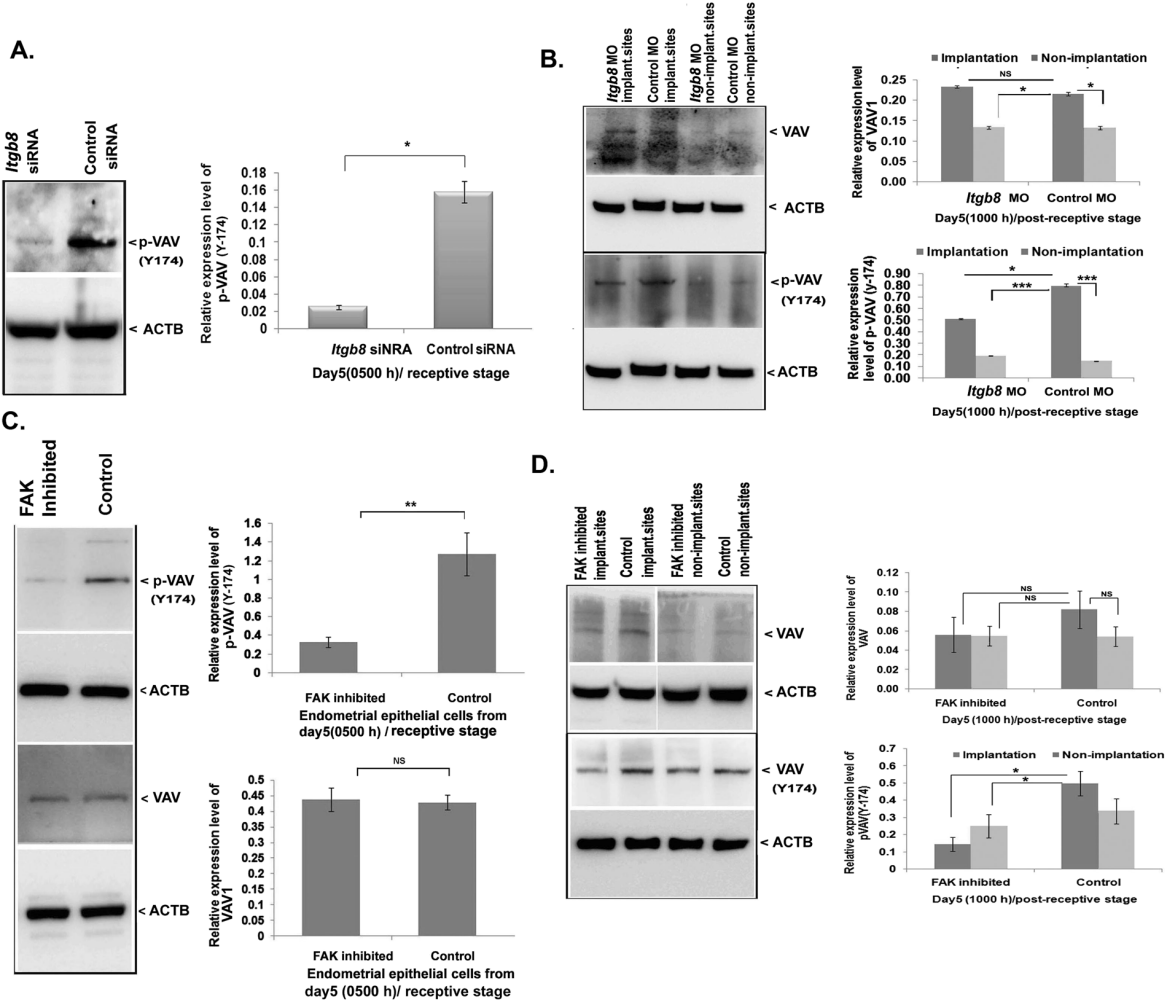
VAV phosphorylation (activity) is regulated by the ITGB8-FAK signaling axis in the epithelial cells of the endometrium during the receptivity phase. (**A**) The activity of VAV was analyzed by western blotting and densitometrically in the endometrial epithelial cells from the receptive stage for embryo implantation. (**B**) Similarly, the expression and activity of VAV were determined in the whole uterine tissue protein fraction (day5, 1000 h) after the *Itgb8* knockdown at day4, 1000 h/pre-receptive stage. (**C**) Later, we analyzed the FAK driven activity of VAV during receptive stage’s endometrial epithelial cells. (**D**) We also investigated the FAK inhibition response on the expression of VAV and its activity in the endometrium (uterus) with the help of immunoblotting and densitometry. (***p < 0.001, *p < 0.05, NS p > 0.05).

We have found that the activity of VAV was decreased when *Itgb8* was silenced in endometrial epithelial cells derived from day5 (0500 h) stage of receptive endometrium period (Fig. 6A). A similar pattern for phosphorylation of VAV at Y174 was noticed at day5 (1000 h) when the endometrial tissue expressed *Itgb8* was silenced using morpholino oligo against it during day4 (1000 h) (Fig. 6B).

### FAK controls the activity (phosphorylation) of VAV in the endometrial epithelial cells

FAK was inhibited using its pharmacological inhibitor in the isolated endometrial epithelial cells (Fig. 4B) and the activity of FAK (phosphorylation) was blocked. Later, we analyzed the VAV’s expression in the isolated endometrial epithelial cells from the uterus at day5 (0500 h) stage. There was no change in the total VAV expression after the pharmacological inhibition of FAK (Fig. 6C). Interestingly, the activity of VAV (a phosphorylated form of VAV at Y174) was reduced drastically (Fig. 6C). Furthermore, the phosphorylated form (active) of VAV was studied post-FAK activity inhibition during day5 (1000 h) in the whole uterus and found decreased (Fig. 6D). The expression level of VAV was not affected in the uterus; however, the phosphorylated form of VAV (Y174) was noticed nearly 3-fold downregulated (Fig. 6D).

### Co-culture of JAr cells spheroids on *ITGB8* silenced/FAK activity inhibited Ishikawa cell monolayer showed the reduced attachment reaction/rate

**Figure 7:**
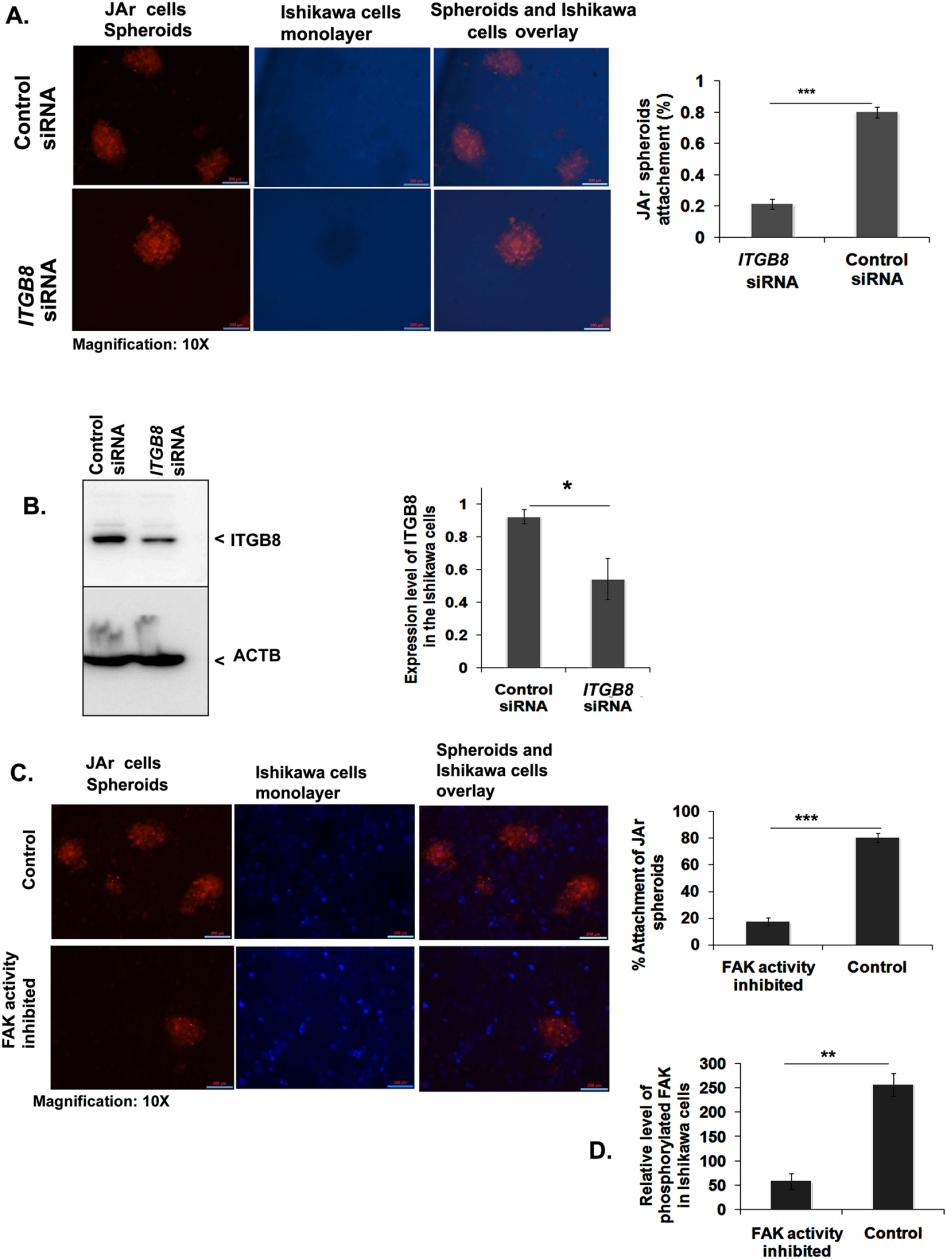
Integrin beta8 and FAK participate in the blastocyst attachment reaction. (**A**) Attachment of human placenta origin JAr cells spheroids on the endometrial epithelial cells (Ishikawa) was analyzed post-*Itgb8* silencing from endometrial cells in percentage. The percentage spheroids adhered/attached were determined after the 24 hrs incubation. (**B**) This was accompanied by the analysis of *Itgb8* knockdown efficiency in the Ishikawa cells by immunoblotting and densitometry. (**C**) Post-FAK activity (tyrosine 397 phosphorylation) inhibition in the Ishikawa cells, JAr cells spheroids attachment were seen and analyzed in the form of the percentage. (**D**) The activity of FAK (phosphorylation) was determined in the Ishikawa cells post-FAK activity inhibition employing ELISA based method. (***p < 0.001, **p < 0.01,*p < 0.05). Scale bars: 200 μm (40X).

To evaluate the ITGB8 or FAK-mediated attachment of blastocyst on the endometrial epithelial cells, we used the spheroids as an embryonic body and co-cultured on the monolayer of the endometrial cells. JAr cells spheroids of 80–100 μm in size were transferred on *IGTB8* depleted (siRNA; 60 nmol) or scrambled siRNA transfected Ishikawa cell monolayer and co-cultured for six hours and the number of spheroids attached was determined (n = 3) (Fig. 7A). Our result showed that the *ITGB8* silenced group demonstrated a reduction in co-cultured spheroids on to endometrial epithelial cell monolayer (Ishikawa cells) (Fig. 7A) and there were nearly 20% adhered JAr spheroids (Fig. 7A). Subsequently, we assayed the ITGB8 protein level in the silenced endometrial epithelial cells and found that nearly 50% *ITGB8* was knockdown by its siRNA from the Ishikawa cells (Fig. 7B) and we confirmed the integrin beta8 siRNA specificity against it (Fig. S3).

Integrin activates focal adhesion kinase (FAK) and mediates molecular signaling^37^. Therefore, further investigation was extended by co-culturing JAr cells spheroids on FAK activity inhibited (30 nM) Ishikawa cell layer. We targeted the inhibition of FAK auto-phosphorylation at the putative activation site, i.e., tyrosine (Y)-397 using FAK Inhibitor 14 (sc-203950). We observed that the inhibition of FAK activity in the Ishikawa cell monolayer significantly reduced the attachment of JAr spheroids by nearly 80% (Fig. 7C). We also determined the activity of FAK by assaying the phosphorylation level at Y397. The phosphorylated-FAK at Y397 was reduced heavily and retained approximately 20% (Fig. 7D).

### Integrin beta8 expression is enhanced when delayed implantation is terminated in response to progesterone and estradiol action

**Figure 8:**
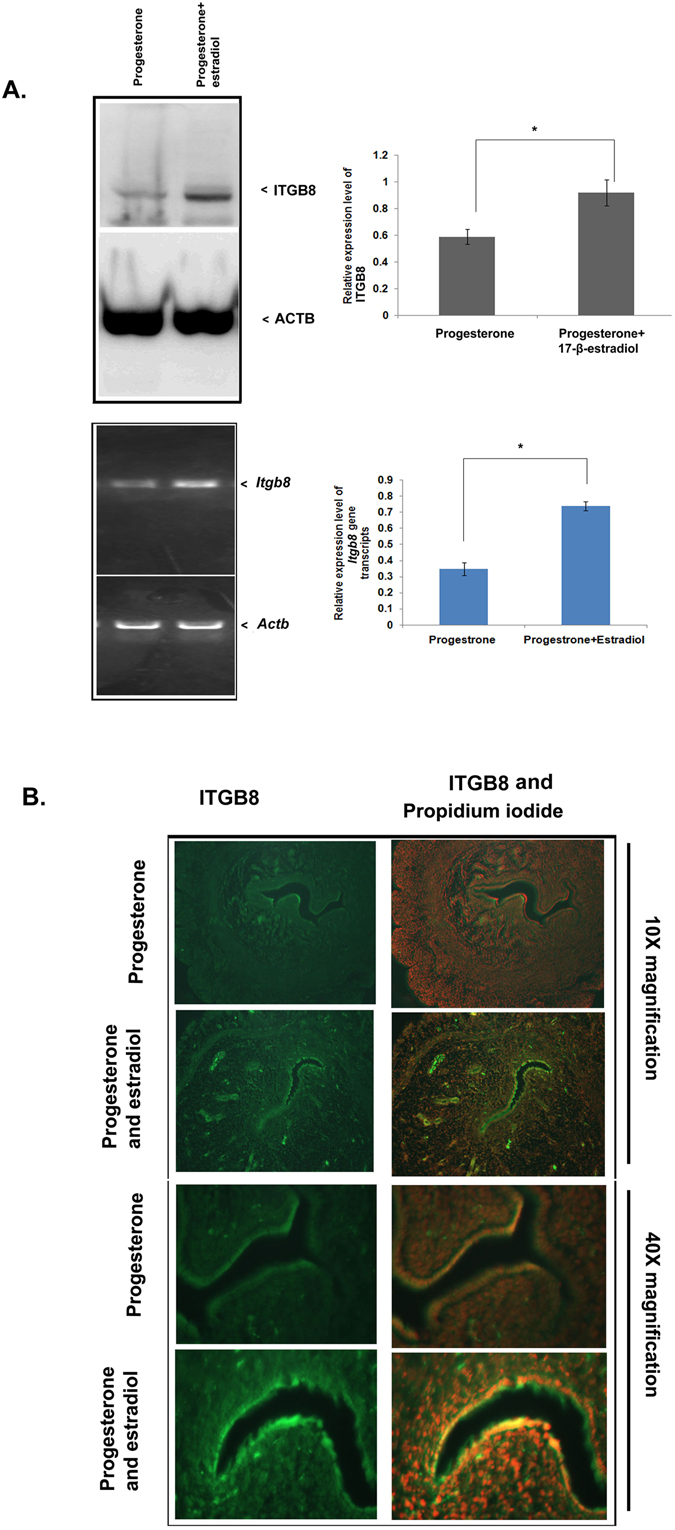
Integrin beta8 is upregulated in the pregnant mouse uterus in response to progesterone and 17-β-estradiol combined supplementation. (**A**) Integrins beta8 expression was analyzed in the pregnant mouse uterus (delayed implantation in a mouse model) using PCR and immunoblotting followed by densitometry. (**B**) The cellular distribution pattern was determined through immuno-fluorescence in the uterine tissue from a delayed implantation mouse model. (*p < 0.05).

Since ovarian steroid governs many of the events of the endometrial receptivity during blastocyst implantation, we determined the expression of ITGB8 in the pregnant uterus during poorly -receptive or delayed receptive/implantation state of the endometrium and activated or receptive state. This domain of study also confirmed the role of progesterone and estradiol-dependent regulation of ITGB8 in the uterus. Our immunoblotting result exhibited a basal level of ITGB8 in the uterus when progesterone (P4) alone was available (Fig. 8A), which surged double of progesterone level in the supplementation of 17-β-estradiol (E2) (Fig. 8A). Similar results were seen in the case of the *Itgb8* transcript level in the uterus (Fig. 8A). Further, we assessed the cell-specific distribution of ITGB8 in the uterus (Fig. 8B) and observed predominant localization of ITGB8 in the endometrial epithelial cells of the glandular and luminal regions in the progesterone alone and there was enhanced fluorescence intensity when the 17-β-estradiol was supplemented (Fig. 8B).

In an earlier report, it has been shown that the down-regulation of ITGB3 happens in the presence of 17-β-estradiol (E2) in the Ishikawa cells, human origin cells^38^. Therefore, we determined the expression level of ITGB8, ITGB3, and RAC1 in response to P4 (1micromolar), E2 (10 nM) and P4 + E2 in the Ishikawa cells. We found that the ovarian steroid administration (progesterone and estradiol) inhibited the expression of ITGB8 and ITGB3 molecules during 24 hrs of treatment (Fig. S4A and B). Interestingly, the expression of total RAC1 was promoted by the P4 or E2, but did not alter by the combination of P4 and E2 (Fig. S4C). Further, the GTP-bound form of RAC1 was increased in the presence of P4, but did not change either by E2 or in combination with P4 and E2 (Fig. S4D).

## Discussion

Integrins in uterus serve both as an anchor as well as a sensor to provide a site for attachment onto endometrial epithelial cell^5, 10, 21, 30^ and inhibition of their functions leads to implantation blockage^10, 30^. Likewise, in earlier finding, we have demonstrated ITGB8 involvement in the endometrial receptivity for the embryo implantation process^10^. The prominent expression of ITGB8 in the endometrial epithelial cells^10^ hints for its involvement in their conduciveness for blastocyst attachment as the epithelial cells prepare for the attachment of hatched blastocyst first. We noticed the upregulated expression of ITGB8 during receptive stage day5 (0500) in the endometrial epithelial cells as well, which can regulate epithelial cell receptivity as the uterine receptivity biomarkers (Phosphorylated STAT-3 and total ITGB3)^21, 30, 39^ were suppressed when *Itgb8* was silenced in the endometrial epithelial cells. This particular data indicates the involvement of ITGB8 in the acquisition of endometrial epithelial receptivity for the blastocyst interaction and attachment reaction. This is also supported by the fact that the uterine receptivity biomarkers, i.e, ITGB3 and STAT3^21, 30, 39^ are found reduced in the recurrent pregnancy loss^40, 41^.

In the receptive endometrium, integrins redistribution and cytoskeleton reorganization can take place in the endometrial epithelial cells during adhesion of implanting blastocyst^4, 21, 26, 42–45^. This hints the mechano-transduction at the cell surface with the help of integrin and ECM, and additionally, that also assists chemical signature amplification intra-cellularily. In addition to integrins serving as a receptor to ECM, they can also propagate the chemical message *via* their associates in the intracellular compartment as integrins lack intrinsic enzymatic activity. Hence, interaction with tyrosine kinases, including FAK is required for integrin-mediated signal transduction^46^.

FAK is one of the downstream signaling molecule of integrin beta and is already reported in the uterine receptivity for blastocyst adhesion^21, 25^. We observed the activation of FAK in response to ITGB8 in the endometrium and endometrial epithelial cell during the receptivity period. In fact, ITGB8 can physically interact with FAK and upregulation of ITGB8 in the implantation sites might induce recruitment of FAK to the apical region of uterine luminal epithelial cells to facilitate the probable conducive environment for a cross-talk between the embryo and endometrium. Focal adhesion proteins (talin, paxillin, and ITGB1) and ITGB3 disassemble from the site of focal adhesions^21, 47, 48^. Therefore, it seems that ITGB8 can function through focal adhesions in the endometrial receptivity development.

In addition to FAK interaction with ITGB8, activation of RAC1 can happen *via* ITGB8^35^ and the RAC1 requirement for the endometrial receptivity is already demonstrated^20, 34^. We found poor activation of RAC1 in absence of *Itgb*8 in the endometrial epithelial cells during acquisition of their receptivity, which confirmed the activation of RAC1 by the ITGB8.

Further, RAC1 is known to be downstream of VAV guanine exchange factor, which is required for small G proteins, RHO/RAC, activity^49^ and VAV is a target of ITGB signaling cascade also where it gets phosphorylated by the integrins^50, 51^. In the present study, we found the diminished activity of VAV (tyrosine phosphorylation) when *Intgb*8 expression was blocked from the day5 (0500 h) stage endometrial epithelial cells and even in the whole uterus during the blastocyst implantation period (day5, 1000 h). This implies that ITGB8 can activate VAV by facilitating the phosphorylation at the tyrosine residue either directly or via associated signaling intermediate like FAK in the endometrial epithelial cells.

Focal adhesion kinase regulated process can facilitate the loose organization of the uterine luminal epithelial cells to become less adherent on the underlying basal lamina facilitating blastocyst invasion^21, 47^. Integrin beta can transduce signaling by associating with the focal adhesions (FAK) complex^52^. Herein, we found FAK levels maximally in the implantation regions of endometrial receptivity stage and similar kind of pattern was also noticed when the activity of FAK was assayed in the endometrium. Surprisingly, the isolated and cultured endometrial epithelial cells mirrored the effect as seen under the *in vivo* conditions, suggesting the acquisition of FAK activity in the development of receptivity of the endometrium. The cellular distribution pattern of FAK was mostly in the apical region during the pre-receptive stage and during the advance stage, the localization of FAK became prominent in the basal region of endometrial epithelial cells, which very well correlated with the earlier observations^32^. Interestingly, FAK inhibition in the uterus during receptivity phase can render the poor implantation of the blastocyst (sites). During an inactive state of FAK in the endometrium, endometrial receptivity/implantation associated biochemical markers, i.e. ITGB3, *Bmp2* and active-STAT3 (phosphorylated, S-727) were compromised in the implantation and non-implantation sites, which confirmed the involvement of FAK in the endometrial receptivity.

FAK undergoes tyrosine phosphorylation^23^ and perform its kinase activity, which in turn regulates the activity of RHOGTPase^53, 54^ as ITGB1 can activate RAC1 *via* FAK^55^. The activities of RAC1 and FAK were found under the control of ITGB8 in the endometrium; however, whether the RAC1 acquires activity in response to FAK-mediated signaling was not answered. Interestingly to note, we found the activity of RAC1 under the FAK regulation in the whole uterus as well as in the endometrial epithelial cells during endometrial receptivity period and confirmed that FAK can receive activation signaling of RAC1 in response to ITGB8.

We found the activity of RAC1 in response to FAK; therefore, we investigated the RAC1 guanine exchange factor, VAV, activation in response to FAK in the endometrial receptivity process. Considering the fact that FAK and VAV interaction can occur in the focal adhesions (FAs)^18^, we found VAV expression unaffected when the FAK activity was inhibited either in the uterus or endometrial epithelial cells. However, the activity of VAV (phosphorylation at tyrosine 174) was seen dependent on the activity of FAK in the uterine implantation and non-implantation regions and the effect was also replicated in the endometrial epithelial cells.

In the latest study, the premature decrease of epithelial apical-basal polarity and defective junction remodeling was seen when the uterine *Rac1* was depleted and in consequence led to disrupted uterine receptivity and implantation failure^20^. RAC1 activity was observed in the endometrial epithelial cells during all the stages of endometrial receptivity; however, it was very prominent during the peri-implantation stage. We gained further insight of RAC1 association with the endometrial receptivity for blastocyst implantation where we found the elevated activity of RAC1 at the blastocyst implantation sites in the uterus. This suggests receptivity specific optimal activity of RAC1 in the endometrium.

Surprisingly, RAC1 silencing was shown to be associated with decreased focal adhesion disassembly and resulted in large focal adhesion complexes in human endometrial stromal cells (hESCs)^19^. This points to the probability of a feedback loop between FAK and RAC1 to control the focal adhesions in the endometrium in association with ITGB8 to facilitate the endometrial epithelial cell receptivity for blastocyst adhesion and establishment of the pregnancy.

Integrins alpha5 beta1 and alpha v beta3 have been reported in the adhesion process of blastocyst^20, 21, 45^. Another member of ITGB family, ITGB8 can also participate in the blastocyst adhesion on endometrial epithelial cells, which is evident by the co-culture of embryonic size JAr cells on the *Itgb8* silenced epithelial cells where depletion of *Itgb8* rendered reduction of spheroid attachment (80%). This particular information suggests that the endometrial epithelial cell expressed ITGB8 can play a direct role in the blastocyst attachment. Integrin beta8 can also propagate the signaling *via* FAK as the functional inhibition of FAK by its autophosphorylation blockage at tyrosine-397 in the endometrial epithelial cells (Ishikawa) leads poor attachment of JAr spheroid similar to the ITGB8. This clearly suggests that ITGB8 activates FAK, which in turn can switch on the VAV-RAC1 signaling axis to bring the endometrial epithelial cell receptivity for blastocyst attachment. Furthermore, the ITGB8 directly interact with the implanting-blastocyst.

During the establishment of endometrial receptivity, the expression of integrin beta8 depends on 17-β-estradiol as the expression level of transcript and protein of ITGB8 was surged when the 17-β-estradiol was available. Interestingly, the epithelial cells of glandular and luminal regions showed most responsiveness towards 17-β-estradiol in the mouse. However, the response of ITGB8 in the human endometrium is quite different as the supplementation of progesterone or estradiol or in combination did not promote ITGB8 expression in the Ishikawa cells. This is similar to ITGB3 expression in response to ovarian steroids as seen in the earlier studies^38, 56^. This cell type is carcinoma in origin and may not represent the normal scenario; therefore, it is difficult to interpret the ovarian steroids response on integrins expression in the Ishikawa cells  with respect to the mouse model system. Interestingly, progesterone (P4) promotes RAC1 expression and activity in the human origin endometrial cells, suggesting that RAC1 activity is modulated by available P4 to coordinate the endometrial receptivity as RAC1 is one of the potential signaling molecules in the uterine receptivity development^20^.

In conclusion, we demonstrated that ITGB8-FAK-VAV-RAC1 signaling axis operates in the endometrial epithelial cells during acquisition of the receptivity to facilitate the blastocyst adhesion for the establishment of the pregnancy. Further, ITGB8 optimal expression is dependent on 17-β-estradiol in the receptive endometrium in particular to epithelial cells during embryo implantation period in the mouse model of pregnancy.

## Experimental Procedure

### Antibodies and Reagents

Progesterone (P0130), 17-β-estradiol (E8875), anti-ITGB8 (HPA027796), Dispase II (D4693), Collagenase Type2 (C6885), anti-ACTB (A2668), anti-Cytokeratin-pan-FITC (F3418), Minimum Essential Medium Eagle (M0894), Non-essential amino acids (M7145), Sodium bicarbonate (S4019), Propidium Iodide (P4170), Protease inhibitor cocktail (S8830) and Phosphatase Inhibitor (P5726) were purchased from Sigma-Aldrich Inc., Bangalore, India. Integrin beta8 siRNA (sc-13138), anti-VAV (cat no. sc132) and FAK inhibitor 14 (sc-203950) were purchased from Santa Cruz Biotechnology Inc., CA, USA. The Immobilon-PVDF membrane (0.2 µm), ECL reagent kits, goat anti-rabbit IgG, HRP conjugate (62114038 0011730) and RIPA lysis buffer (20188) were purchased from Merck-Millipore, Bangalore, India. Antibody against RAC1 (ab33186) and Phosphorylated-VAV (Y174) (ab47282) were purchased from Abcam, Cambridge, MA, USA. Nonfat milk (170–6404), protein assay kit (500–0116) and DMEM/F12 (12634010 and 21021025), FBS (10082-147), Lipofectamine, OptimMEM (31985070), Cell Tracker CMF_2_HC Dye (C12881), Cell Tracker Red CMTPX (C34552), Oregon Green 488 goat anti-rabbit IgG (H + L) (O6381), PCR Super Mix (12580-015), SuperScript III cDNA synthesis kit (18080-126 400), and antibiotics and antimycotics (154240-016) were obtained from ThermoFisher Scientific, Bangalore, India. The G-LISA RAC1 activation assay Biochem Kit (BK128) was purchased from Cytoskeleton, Denver, CO, USA. Antibodies against FAK (3285 S) and phosphorylated-FAK (Tyr397) (D20B1) Rabbit mAb (8556) were purchased from Cell Signaling Technology Inc., Danvers, MA, USA.

### Pregnancy animal model

Female mice (*Mus musculus﻿;* Swiss albino strain) were housed in a temperature-controlled and light (12.00–12.00)/dark cycle controlled room. Water and food were available ad libitum during the experiment. The study was approved by the institutional ethical committee on animal experiments (IAEC) at Council of Scientific and Industrial Research (CSIR)-Central Drug Research Institute (CDRI), Lucknow, India (IAEC/2013/46 dated 29^th^ May 2013) and experiments were conducted at CDRI Laboratory Animal Facility in accordance with the guidelines approved IAEC at CSIR-CDRI, Lucknow, India. The animals were sacrificed on different days of embryo implantation, i.e., day4 (1000 h) (pre-implantation), day4 (1600 h) (late pre-implantation), day5 (0500 h) (peri-implantation) and day5 (1000 h) (post-implantation) and uterine tissue was collected as described previously, and embryos were examined microscopically (TS100-F Inverted Phase Contrast Microscope, Nikon, Japan) to determine the various stages of receptivity.

### Murine uterine epithelial cells isolation, culture, FAK inhibition and transient knockdown assay

This protocol is already optimized in our setting^57^. After washing with PBS, fat and connective tissues were removed and the uterus was sliced longitudinally. The sliced uteri were incubated in PBS supplemented with 0.1% collagenase type2^58–60^ and dispase-2^61^ at 37 °C for 10 min and the supernatant was transferred into a 15 ml tube containing 2 ml FBS. This step was repeated four times and finally, the supernatant was centrifuged at 2000 rpm for 5 minutes. Then the yield of epithelial cells was obtained using EasySep™ Mouse Epithelial Cell Enrichment Kit (19758, Stem Cells Technologies Inc. Vancouver, Canada) following the manufacturer’s instructions^57^. After enrichment using EasySep kit, the mouse endometrial epithelial cells mixed and cultured in 10% fetal bovine serum (FBS), 1% antibiotics supplemented DMEM, and this time point was considered as ‘0 hour’. After 12 hours, for transient knockdown of *Itgb8*, the cells were starved for next 4 hours. Subsequently, transfection of siRNA (60 pmoles siRNA using Lipofectamine reagent) in the OptiMEM medium was done for 12 h. Later, the culture medium was replaced with 10% fetal bovine serum (FBS) and 1% antibiotics supplemented DMEM and incubated further for 12 hrs (total knockdown time was 24 hours). Finally, cells were washed in PBS before use for analysis of protein.

In the context of FAK activity inhibition, endometrial epithelial cells were cultured for 24 hrs, followed by FAK drug inhibitor (20 µM) treatment for next 24 hrs. Thereafter, cells were washed in PBS and harvested to prepare cell lysate and quantification.

### Immuno-fluorescence

Mouse uterine epithelium cells were seeded at 2 × 10^6 ^cells/well in a 6-well dish containing sterile coverslips. After 24 h, cells were fixed with 4% paraformaldehyde in PBS (20 min at 37 °C). Next, cells were washed with PBS and permeabilized with 0.2% Triton X-100 and 1% BSA in PBS (20 min at room temperature). Cells were again washed with PBS and then blocked with 1% BSA in PBS for 2 hours at room temperature and then incubated with Anti-Cytokeratin pan-FITC for overnight at 4 °C. Finally, cells were washed three times with PBS and DAPI (1 µg/ml) was used for nuclear stain. Coverslips were mounted onto slides using 70% glycerol^57^. Images were acquired using a laser scanning confocal microscope (Carl Zeiss LSM 510 META, Jena, Germany) equipped with a plan apochromat 63x oil/1.4 NA DIC objective, and 3x optical zoom over 63x objective (189 X).

### Immuno-histochemistry of ITGB8 and FAK

The immunolocalization study was done according to the earlier described method with some minor modification^61^_._ The 5 μm sections of tissue were deparaffinized overnight (12–15 hr) in xylene and allowed to rehydrate with subsequent changes of the gradient of ethanol (100%, 95%, 70%, and 50%) and distilled water for 10 min with two changes. Antigen retrieval was performed in sodium citrate buffer (10 mM, pH 6) for 20 min followed by the immuno-fluorescence method as described previously^10, 57^. Tissue sections were further blocked in 5% goat serum for 1 hr and subsequently, after washing with PBS, incubated overnight at 4 °C with the ITGBβ8, and FAK antibodies in 1:100 dilutions and normal rabbit IgG in 1/100 dilutions in 2% goat serum. For the immuno-fluorescence, anti-rabbit FITC conjugated antibody was incubated and counterstained with the Propidium Iodide (PI)^10^. Slides containing tissue sections were dehydrated using ethanol gradient (50%, 70%, 90%, 100%) and absolute xylene. Finally, uterine tissue sections were mounted using either DPX or 50% glycerol and the image were captured using a phase contrast inverted phase contrast microscope (CKX41 Trinocular with Cooled CCD Camera Model Q Imaging MP5.0-RTV-CLR-10-C from Olympus, Tokyo, Japan).

### *ITGB8* esiRNA transfection and FAK activity inhibition in the human endometrial cells (Ishikawa cells)

Ishikawa cells (Human endometrial adenocarcinoma epithelial cell line) was a kind gift from Dr. Anila Dwivedi, Endocrinology division, (CSIR-CDRI, Lucknow, India) and was maintained in Minimum Essential Medium Eagle supplemented with 1% non-essential amino acids, and 2.2 gm/L sodium bicarbonate and 1% anti-mycotics and antibiotics supplemented with 10% fetal bovine serum. Cells were grown at 37 °C and 5% CO_2_ in humidified condition.

JAr cells [HTB-144, American Type Culture Collection (ATCC), Manassas, VA, USA], a human choriocarcinoma cell line, were maintained in advanced DMEM/F12 supplemented with 10% FBS and 1% anti-mycotics and antibiotics grown at 37 °C and at 5% CO_2_ concentration in humidified condition.

Ishikawa cells were seeded in a 12-well culture plate (~5 × 10^3^ cells per well) for 12 h in a CO_2_ incubator in humidified condition. The cells were incubated with serum-free media for 4 h before the esiRNA transfection. The esiRNA against *ITGB8* (n = 3) (EHU014221, Sigma-Aldrich Inc.), targeting sequence is, 5′TCCAGAATGTGGATGGTGTGTTCAAGAG GATTTCATTTCAGGTGGATCAAGAAGTGAACGTTGTGATATTGTTTCCAATTTAA TAAGCAAAGGCTGCTCAGTTGATTAAATACCCATCTGTGCATGTTATAATACCCA CTGAAAATGAAATTAATACCCAGGTGACACCAGGAGAAGTGTCTATCCAGCTGCGTCCAGGAGCCGAAGCTAATTTTATGCTGAAAGTTCATCCTCTGAAGAAATATCCTGT3′ or control siRNA (The Sigma-Aldrich MISSION siRNA; Universal Negative Control, SI001.). There was 1 µl of each esiRNA at a concentration of 50 ng/µl and was mixed with 50 µl of Opti-MEM in RNase-free microcentrifuge tube separately. At the same time, a suspension of 1 µl of lipofectamine with 50 µl of OptiMEM in RNase-free tube was prepared and incubated for 20 min at room temperature. Further, the Opti-MEM-esiRNA suspension and lipofectamine-Opti-MEM-suspension were mixed with each other followed by incubation for another 30 min at room temperature and then added to the wells containing a serum free media (900 µl). Cells were incubated with 5% CO_2_ and 37 °C, in humidified condition, for 12 h. Media was changed after 12 h and grown in complete media for another 12 h.

For FAK activity inhibition, when the cell reached 60–70% confluence, the cells were treated with FAK inhibitor at a concentration of 30 nM and another 3 wells were served as a control (n = 3). Cells were grown further for 24 h in an incubator with 5% CO_2_ and 37 °C, in humidified condition. After 24 h incubation, the cells were stained with live cell tracker, Cell Tracker Blue CMF_2_HC Dye prepared in PBS for 30 min and it was replaced with DMEM/F-12, without phenol red.

### *In vitro* embryo implantation by JAr spheroids co-culture on endometrial epithelial cell monolayer

The assay was performed according to the previous method^62^. The JAr cells spheroids were prepared in DMEM, F-12 medium. Afterwards, the cells were collected after trypsinization in DMEM/F-12 media and the flask was incubated on dancing shaker with 200 rpm, at 37 °C and at 5% CO_2_ concentration in humidified condition for 12 h. Then spheroids were filtered through 100 µm membrane and the filtrate was re-filtered using 70 µm membrane to collect spheroids having size between 70–100 µm in size. These spheroids were incubated for 10 min with live cell tracker dye Cell Tracker Red CMTPX, and re-suspended in DMEM/F-12, without phenol red. The spheroids were co-cultured on *ITGB8* siRNA, FAK inhibitor or respective control treated monolayer of Ishikawa cells for 10 min and washed with white media/DMEM/F-12, without phenol red and the attached spheroids were counted and imaged using microscope (CKX41 Trinocular with Cooled CCD Camera Model Q Imaging MP5.0-RTV-CLR-10-C from Olympus, Tokyo, Japan).

### Intraluminal delivery of MO to silence the endometrial *Itgb8*

This is accordance with our earlier published method^10^. We received the morpholino oligonucleotides sequence (GGCCGAGCCGCACATAATGCAAAGC, 25 mer) against the mouse *Itgb8* target sequence (﻿in bracket)﻿﻿; ﻿GGTGCGCTCTCAAGTCGGGAGAGCGCATCAGGAAAGCAACTAGGCAG TAGGCACCGGGCGGGCT[GCTTTGCATT(ATG)TGCGGCTCGGCC]CTGGCTTTTC (NM_177290.3, *Mus musculus integrin beta8* (*Itgb8*), mRNA) from Gene Tool, Philomath, OR, USA, which does not overlap with other integrins sequence such as *Itgb*3 or *Itgb6* sequences through BLAST. We have already published one study on this given MO-based silencing of *iItgb8* ^10^. The pregnant female mice underwent a mini-laparotomy under anesthesia at day4 (1000 h) of the window of uterine receptivity to deliver the *Itgb8* MO (2 µl of 20 nM) in one of the horn, while the other horn of the same animal (control) received (2 µl of 20 nM) control MO as reported earlier^10^. After the Evans blue dye injection, the animals were sacrificed on day5 (1000 h) for the study of embryo implantation sites during post-implantation [day5 (1000 h)]. The uterus was photographed to record the number of implanted embryos.

### Treatment of pregnant mice with FAK inhibitor

The pregnant female mice on the day4 (1000 h) underwent a mini-laparotomy as mentioned in the previous section. One uterine horn of mouse was injected with 2 µl of FAK inhibitor (20 µM) into the lumen through the oviductal site, while another horn was injected with 2 µl of water and served as a control as described elsewhere^10^. Implantation sites and inter/non-implantation sites in the uterus were visualized as per earlier mentioned method^10^.

### Delayed implantation mouse model

We adopted previously described protocol^57^ where pregnant female mice were bilaterally ovariectomized on day3 (1600 h) in the presence of anesthetics. Progesterone at 1.0 mg/25 gm of body weight (50 μl/animal) dose was delivered to animals *via* a subcutaneous route at 5:00 pm during 3^rd^ until the 7^th^ day of pregnancy whereas the17-β-estradiol (25 ng/25 gm of body weight) was administered only on day 7 of pregnancy.

Uterus was collected at 1000 h on the 8^th^ day of pregnancy and embryos were recovered to verify the pregnancy status (delayed or activated) as described previously^57^. Tissues were processed for protein extraction and quantification^57^.

### Polymerase chain reaction (PCR)

Using Trizol reagent, total RNA was extracted in RNase-free condition from the uterine tissue of the window of implantation/receptivity period and post-*Itgb*8 knockdown as described previuosly^57^. The cDNA was synthesized individually (n = 5) using 1 µg RNA and SuperScript III cDNA synthesis kit as per the given instructions provided by the manufacturer. PCR was performed according to earlier described methods^10, 63^ with minor modifications. PCR reaction for each sample consists of cDNA (0.25 µg), 23 µl of Platinum blue PCR Super Mix and 20 pmol of forward and reverse primers. PCR was performed on a C1000 thermal cycler (Bio-Rad Laboratories, CA, USA) for 38 cycles (95 °C for 1 min; 53 °C for 1 min, 72 °C for 1 min and a final extension for 10 min at 72 °C) with one initial incubation (denaturation) at 95 °C for 5 min. Actin beta (*Actb*) was used as an endogenous control as described in the earlier study^64^. PCR primers of *Itgb8* ^10^, *Fak*, *Rac1*, *Bmp2 and Actb* were synthesized by Integrated DNA Technology (IDT), Belgium. Amplified products were resolved on 1.5% agarose gel and imaged using the Gel Documentation system (Bio-Rad, Hercules, CA, USA). Band intensity was quantified using Total Lab Quant 1D gel analysis software version 5.01 (Nonlinear Dynamics Ltd., Newcastle upon Tyne, U.K.).

### Protein extraction

The uterine tissue was gently washed with buffer (pH7.4) containing100-mM KCL, 3-mM NaCl, 3.5-mM MgCl_2_, 10-mM Pipes, phosphatase inhibitor cocktail, and protease inhibitor cocktail as reported previously^57^. Tissue was homogenized followed by centrifugation at 200 x g (4 °C) for 10 min and the supernatant was centrifuged again at 1,500 x g for 10 min and the resultant post-nuclear supernatant was again centrifuged at 12,000 x g for 10 min. The obtained post-mitochondrial supernatant was used as whole uterine tissue crude cytosolic and membrane combined protein extract and was stored at −80 °C for further use^57^.

Whole cell lysates of Ishikawa and isolated primary epithelial cells were prepared with cell lysis buffer (RIPA). Protein concentration was estimated by Pierce BCA Protein Assay kit with bovine serum albumin as standard.

### SDS-PAGE, Western blotting and immunoblotting

Proteins were denatured with Laemmli buffer^65^ containing β-mercatpoethanol. The sample containing 20 μg protein was separated on a 10% SDS-PAGE under denatured condition^66^. The blotted membranes were blocked overnight with 5% nonfat milk for 2 hrs, followed by incubation with respective primary antibodies against ITGB8 (dilution 1:1000)^10^, FAK (dilution1:1000), VAV and p-VAV (dilution 1:1000), RAC1 (dilution 1:3000), and ACTB (dilution 1:8000) at 4 °C temperature. Finally, membranes were probed with respective secondary antibodies (anti-rabbit IgG or anti-mouse IgG HRP) in 1:3000 dilution. Immuno-blot band intensity was analyzed by TotalLab Quant (Nonlinear Dynamics, Newcastle Upon Tyne, UK).

### RAC1 activity assay

The RAC1 activity assay was performed as per the method described earlier^67^. Briefly, a total of 60 µg protein extracts were used in respective wells that were pre-coated with RAC-GTP-binding protein. Subsequently, 30 min incubation was allowed at 4 °C followed by successive incubation with 50 µl of anti-RAC1 for 45 min. Next, 50 µl secondary antibody (conjugated with HRP) was added for 45 min. Color development was done using 50 µl of HRP detection reagent and incubated for another 20 min. The reaction was stopped by the addition of HRP stop solution (50 µl) provided in the kit. Optical density was recorded at 490 nm using a microplate spectrophotometer.

### FAK activity (Y-397) measurement by ELISA

FAK is activated by its phosphorylation at tyrosine (Y-397). Here, its activity was measured using a sandwich ELISA kit (DYC4528E, R&D Systems, Inc. MN, USA.) as per manufacturer protocol. A total 40 µg of protein extract was added to a respective well that was pre-coated with the FAK-specific antibody (PTK2). Subsequently, blocked with 1% BSA for 1 h. Samples were added to the respective wells and incubated further 2 h at room temperature. The streptavidin-HRP conjugate was added to each well and incubated for 20 min followed by color development using HRP substrate for another 20 min. The reaction was terminated by addition of stop solution and absorbance was measured at 450 nm using microplate spectrophotometer (iMARK microplate reader, BioRad Laboratories, CA, USA).

### Immunoprecipitation

Protein extracts from different stages of uterine receptivity were precleared with Protein A-Agarose according to previously described method^10^. The pre-cleared protein extract was incubated with anti-ITGB8 (5 µg/100 µg) in 100 µg protein extract overnight at 4 °C. The Protein A-Agarose was washed in PBS, and 50% Protein-A Agarose slurry was prepared in the cytosolic buffers. The protein complexes were captured by the addition of Protein-A-Agarose (10 µg/100 µg) for 2 h at 4 °C. Thereafter, IP protein samples were resolved by 12% and 10% SDS-PAGE and subjected to immunoblotting.

### Statistical analysis

All the experiments were performed for a minimum of three to five times using separate individual animals (mouse; n = 3/5) as replicates. Protein band intensities from blots were averaged and the standard error of the mean (s.e.m) was calculated and results are shown as mean ± s.e.m. Group comparisons were made using Student’s *t-*test with a significant difference assignment at p < 0.05. The results of western blot band intensity are shown as a ratio (protein of interest/β-actin) to correct loading error for each sample.

## Electronic supplementary material

Supplemental information
