# Supplementary material for: Integrin beta8 (ITGB8) activates VAV-RAC1 signaling via FAK in the acquisition of endometrial epithelial cell receptivity for blastocyst implantation
Source: Sci Rep. 2017 May 15;7:1885. doi: 10.1038/s41598-017-01764-7 (PMC5432530; doi:10.1038/s41598-017-01764-7)
Supplement: Supplementary file 1 — Supplemental information [file 41598_2017_1764_MOESM1_ESM.pdf]

Supplemental information (Figures, and Materials and Methods)

SREP-16-46271

DOI : 10.1038/s41598-017-01764-7

Title: Integrin beta8 (ITGB8) activates VAV-RAC1 signaling via FAK in the acquisition of endometrial epithelial cell receptivity for blastocyst implantation.

Authors: Vijay Kumar[1], Upendra Kumar Soni[1], Vineet Kumar Maurya[1], Kiran Singh [2], Rajesh Kumar Jha [1#].

Affiliation: [1]Division of Endocrinology, CSIR-Central Drug Research Institute, Sector-10, Jankipuram Extension, Sitapur Road, Lucknow-226031, U.P., India. Phone +91-7376818092. [2]Department of Molecular & Human Genetics, Banaras Hindu University (BHU), Varanasi, U.P., India.

[#]**Corresponding author:**

Rajesh Kumar Jha, Ph.D.,

Scientist,

Division of Endocrinology,

CSIR-Central Drug Research Institute,

Life Science North101/111B,

BS 10/1, Sector-10, Jankipuram Extension,

Sitapur Road, Lucknow-226031, U.P., India.

Phone +91-7376818092, e-mail: rajesh_jha@cdri.res.in,

Web page: http://www.cdriindia.org/rdivision5.htm

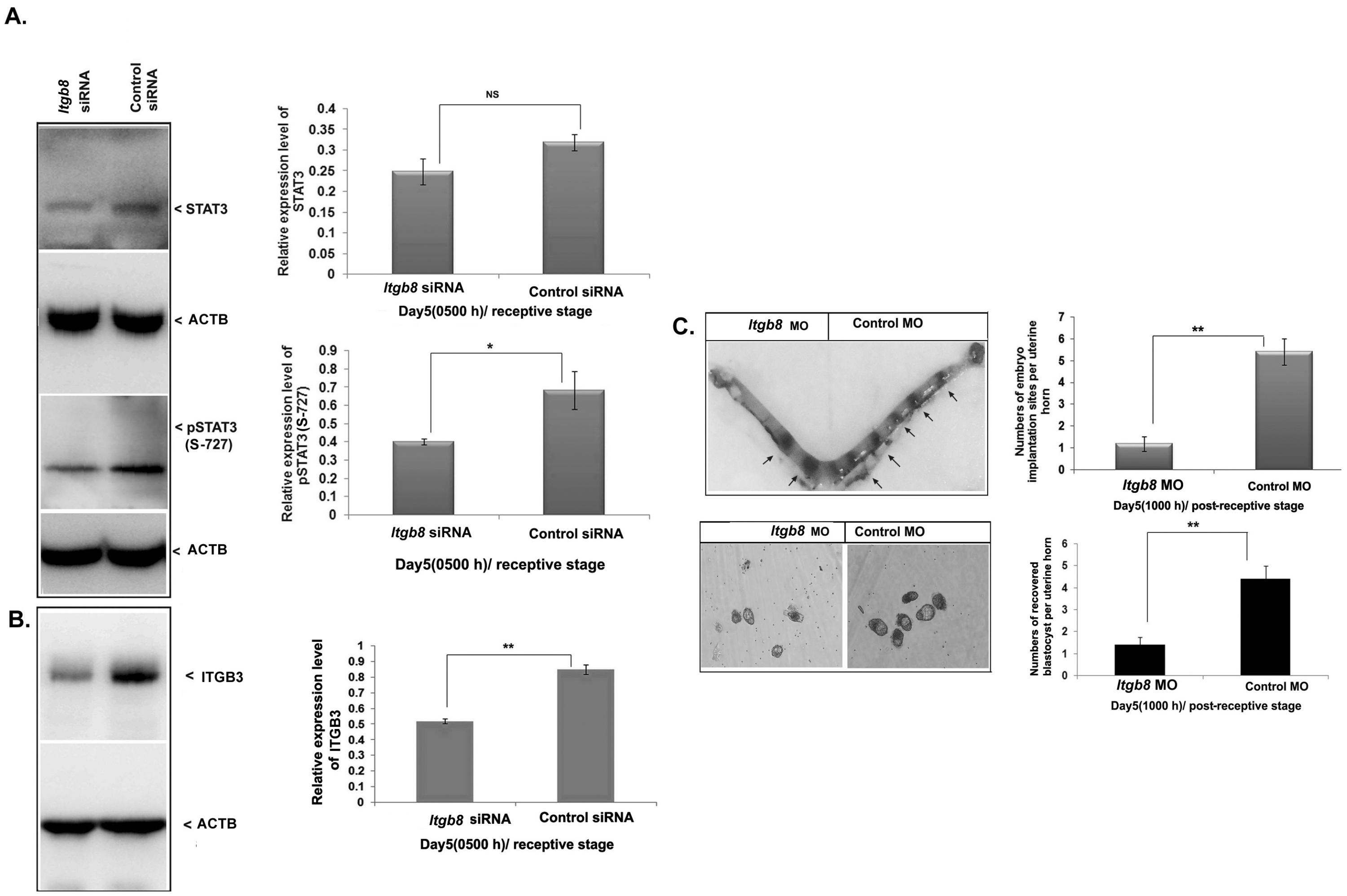

A.
Itgb8 siRNA
Control siRNA
< STAT3
< ACTB
< pSTAT3 (S-727)
< ACTB
NS
Relative expression level of STAT3
Itgb8 siRNA
Control siRNA
Day5(0500 h)/ receptive stage
*
Relative expression level of pSTAT3 (S-727)
Itgb8 siRNA
Control siRNA
Day5(0500 h)/ receptive stage
B.
< ITGB3
< ACTB
**
Relative expression level of ITGB3
Itgb8 siRNA
Control siRNA
Day5(0500 h)/ receptive stage
C.
Itgb8 MO
Control MO
**
Numbers of embryo implantation sites per uterine horn
Itgb8 MO
Control MO
Day5(1000 h)/ post-receptive stage
Itgb8 MO
Control MO
**
Numbers of recovered blastocyst per uterine horn
Itgb8 MO
Control MO
Day5(1000 h)/ post-receptive stage

Figure-S1.

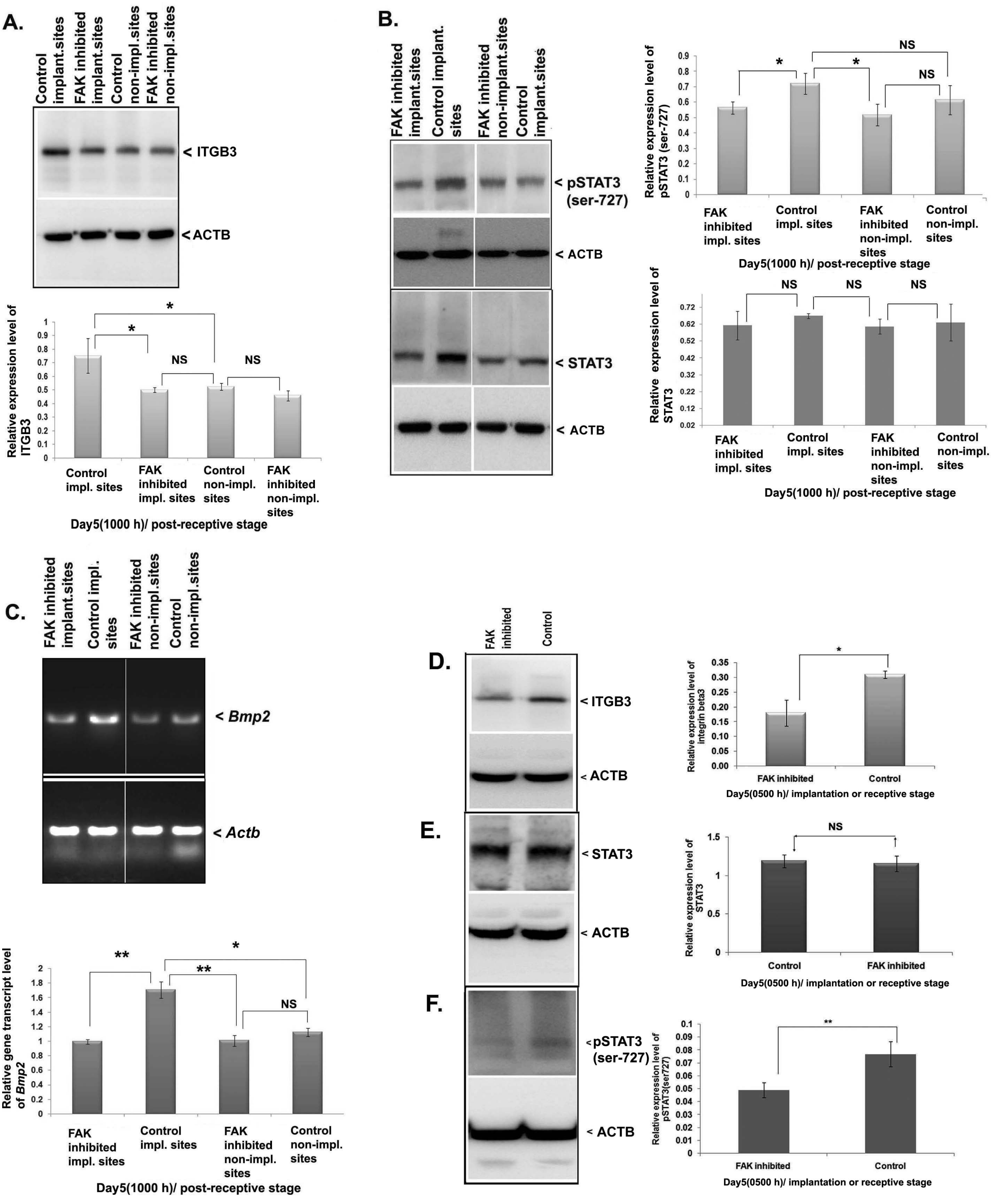
A.
Control implant.sites
FAK inhibited implant.sites
Control non-impl.sites
FAK inhibited non-impl.sites
< ITGB3
<ACTB
Relative expression level of ITGB3
*
*
NS
NS
Control impl. sites
FAK inhibited impl. sites
Control non-impl. sites
FAK inhibited non-impl. sites
Day5(1000 h)/ post-receptive stage
B.
FAK inhibited implant.sites
Control implant. sites
FAK inhibited non-implant.sites
Control implant.sites
< pSTAT3 (ser-727)
< ACTB
< STAT3
< ACTB
Relative expression level of pSTAT3 (ser-727)
NS
*
*
NS
FAK inhibited impl. sites
Control impl. sites
FAK inhibited non-impl. sites
Control non-impl. sites
Day5(1000 h)/ post-receptive stage
Relative expression level of STAT3
NS
NS
NS
FAK inhibited impl. sites
Control impl. sites
FAK inhibited non-impl. sites
Control non-impl. sites
Day5(1000 h)/ post-receptive stage
C.
FAK inhibited implant.sites
Control impl. sites
FAK inhibited non-impl.sites
Control non-impl.sites
< Bmp2
< Actb
Relative gene transcript level of Bmp2
**
*
**
NS
FAK inhibited impl. sites
Control impl. sites
FAK inhibited non-impl. sites
Control non-impl. sites
Day5(1000 h)/ post-receptive stage
D.
FAK inhibited
Control
< ITGB3
< ACTB
Relative expression level of integrin beta3
*
FAK inhibited
Control
Day5(0500 h)/ implantation or receptive stage
E.
< STAT3
< ACTB
Relative expression level of STAT3
NS
Control
FAK inhibited
Day5(0500 h)/ implantation or receptive stage
F.
<pSTAT3 (ser-727)
< ACTB
Relative expression level of pSTAT3(ser727)
**
FAK inhibited
Control
Day5(0500 h)/ implantation or receptive stage

Figure-S2.

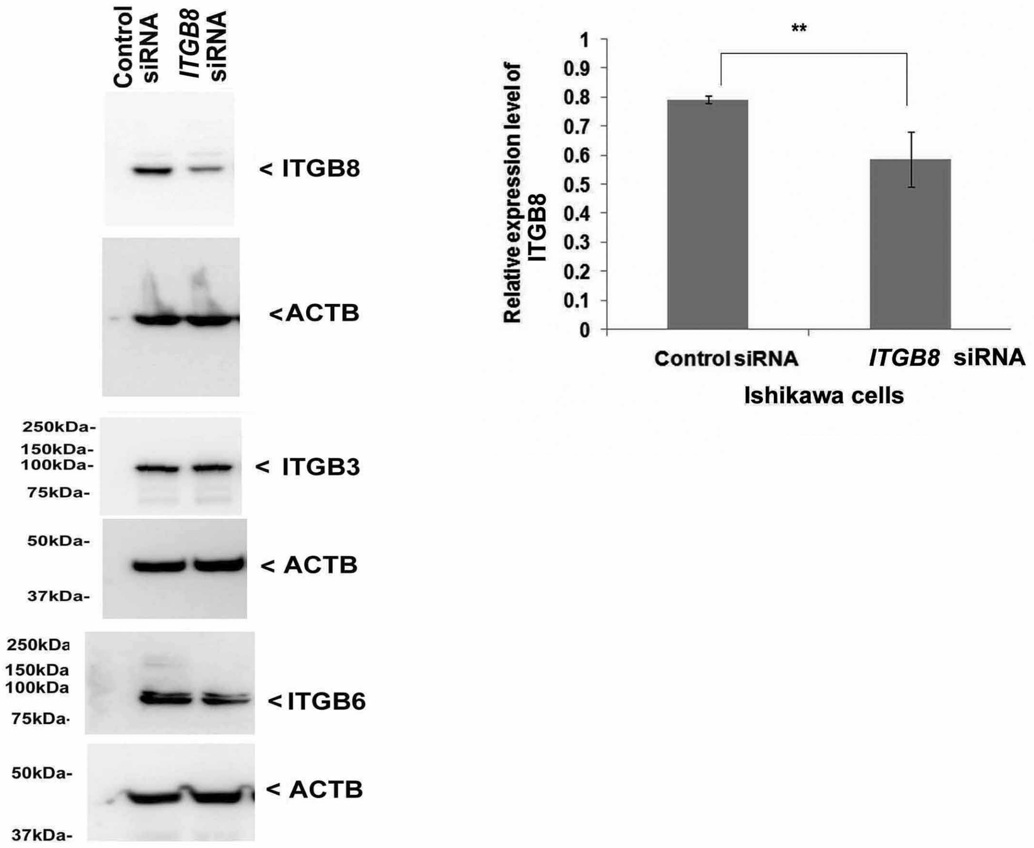
Control siRNA
ITGB8 siRNA
< ITGB8
<ACTB
250kDa-
150kDa-
100kDa-
75kDa-
< ITGB3
50kDa-
37kDa-
< ACTB
250kDa
150kDa
100kDa
75kDa-
<ITGB6
50kDa-
37kDa-
< ACTB
Relative expression level of ITGB8
1
0.9
0.8
0.7
0.6
0.5
0.4
0.3
0.2
0.1
0
**
Control siRNA
ITGB8 siRNA
Ishikawa cells

Figure-S3.

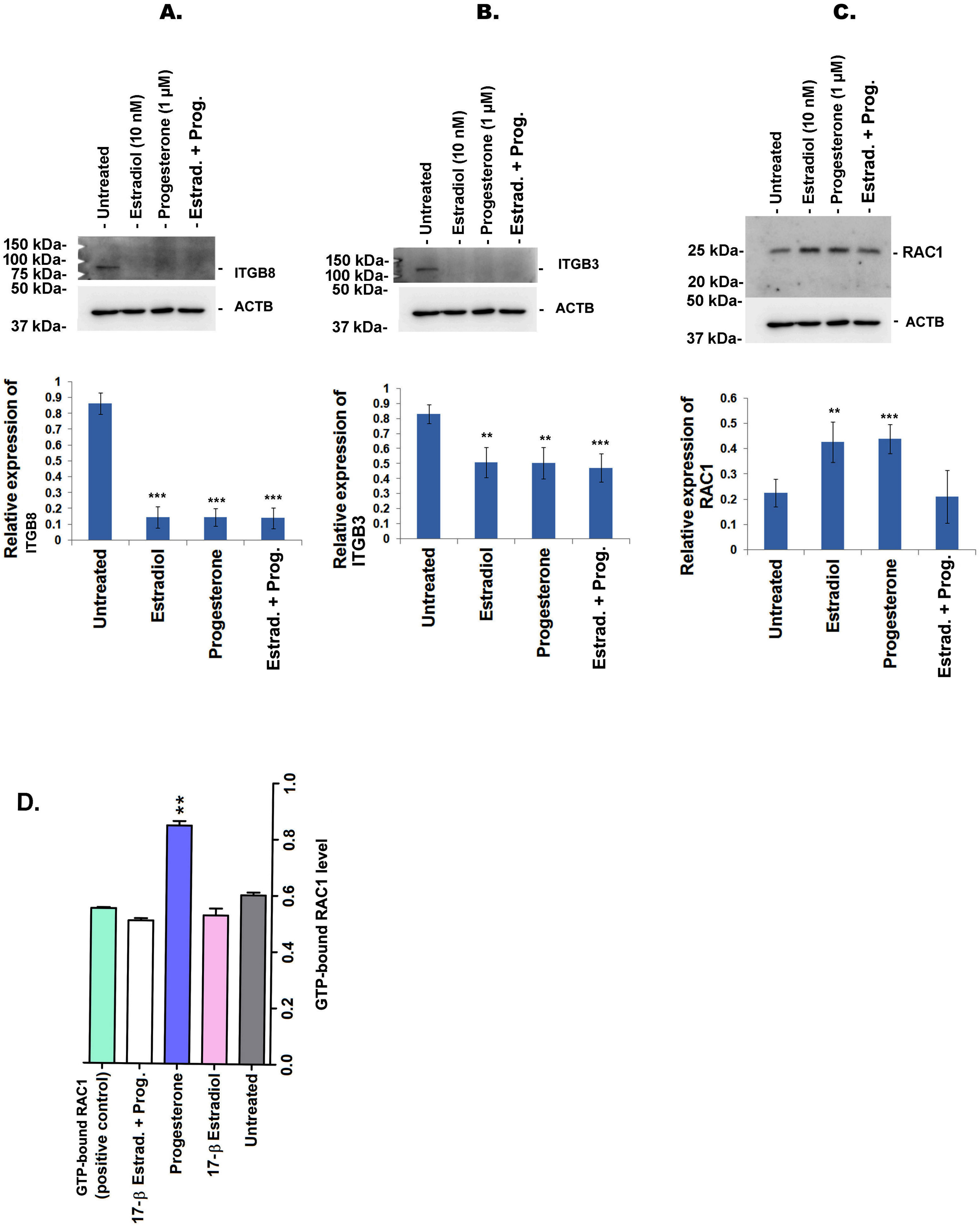
A.
- Untreated
- Estradiol (10 nM)
- Progesterone (1 μM)
- Estrad. + Prog.
150 kDa-
100 kDa-
75 kDa-
50 kDa-
37 kDa-
- ITGB8
- ACTB
Relative expression of ITGB8
Untreated
Estradiol
Progesterone
Estrad. + Prog.
B.
- Untreated
- Estradiol (10 nM)
- Progesterone (1 μM)
- Estrad. + Prog.
150 kDa-
100 kDa-
50 kDa-
37 kDa-
- ITGB3
- ACTB
Relative expression of ITGB3
Untreated
Estradiol
Progesterone
Estrad. + Prog.
C.
- Untreated
- Estradiol (10 nM)
- Progesterone (1 μM)
- Estrad. + Prog.
25 kDa-
20 kDa-
50 kDa-
37 kDa-
- RAC1
- ACTB
Relative expression of RAC1
Untreated
Estradiol
Progesterone
Estrad. + Prog.
D.
GTP-bound RAC1 level
GTP-bound RAC1 (positive control)
17-β Estrad. + Prog.
Progesterone
17-β Estradiol
Untreated

Figure-S4

Figure-S1: Integrin beta8 regulates embryo implantation associated biochemical markers in the endometrial tissue and endometrial epithelial cells in the mouse model. (A) Integrin beta8 silencing from mouse endometrial epithelial cells post 24 hrs culture from day5 (0500 h) stage caused poor phosphorylation of STAT3 (serine 727). (B) Transient knockdown of *Itgb8* from the mouse endometrial epithelial cells (day5, 0500 h) led down-regulation of expression of ITGB3. (C) Transient silencing of *Itgb8* from one of the mouse uterine horns at pre-implantation or day4 (1000 h) stage showed the reduction of implantation sites and blastocyst numbers (recovered). Actin-beta was used as loading control to normalize the immunoblot values. (** $p<0.01$,*$p<0.05$, NS $p>0.05$).

Figure-S2: FAK affected the embryo implantation related biomarkers, i.e., ITGB3, STAT3, and *Bmp2* signaling in the process of mouse endometrial epithelial cell receptivity. (A) The expression level of ITGB3 was determined using immunoblotting in mouse endometrium during day5 (1000 h) after the FAK activity inhibition at day4 (1000 h). (B) Similarly, the expression of total and phosphorylated forms of STAT3 was determined by immunoblotting in the mouse endometrium at day5(1000 h) /post-implantation stage after the FAK activity inhibition. (C) PCR analysis of *Bmp2* was done in the mouse uterus post-FAK activity inhibition during day5(1000 h) of window of endometrial receptivity. (D) Later, the expression of ITGB3 was assayed using immunoblotting in the mouse endometrial epithelial cells after the FAK activity inhibition. (E and F) Similarly, the expression level of total and phosphorylated forms of STAT3 was assayed after the FAK activity inhibition in the endometrial cells from day5, 0500 h stage. Actin beta was used as a loading control to normalized the immunoblot values. (** $p<0.01$,*$p<0.05$, NS $p>0.05$).

Figure-S3: Silencing RNA against *ITGB8* found to be specific for its target and did not have an off-target effect. Integrin beta8 was silenced in the human endometrial epithelial cells (Ishikawa) and we determined the expression level of ITGB8 along with other beta integrins (ITGB3 and ITGB6). Post-*ITGB8* silencing in the Ishikawa cells, the expression of ITGB3 and ITGB6 was detected by immunoblotting and found unchanged when compared with the scrambled siRNA. (** $p<0.001$).

Figure-S4. Integrins beta3 and beta8 were seen downregulated in the human origin endometrial cells (Ishikawa cells) in response to either progesterone or 17-β-estradiol alone or in combination with progesterone and 17-β-estradiol supplementation.

However, RAC1 was seen upregulated in progesterone or 17-β-estradiol supplementation, but not in progesterone and 17-β-estradiol combined supplementation. (A, B and C) Using immunoblotting and densitometry, the expression analysis of ITGB3, ITGB8, and RAC1 was done post-progesterone (P4), 17-β-estradiol (E2), and progesterone and 17-β-estradiol combined treatment in the Ishikawa cells during 24 hrs of culture. (D) The level of GTP-bound form of RAC1 was determined biochemically in response to progesterone, 17-β-estradiol, and progesterone and 17-β-estradiol combined supplementation (24 hrs) in the Ishikawa cells. (***p<0.0001, ** p<0.01, NS p>0.05).

Supplementary Materials and Methods

Steroidal treatment to Ishikawa cells: Ishikawa (Human endometrial epithelium carcinoma cells) cells were grown in a minimum essential medium as described earlier[1]. After reaching cells confluency at 80%, cells were treated either with estradiol (10 nM), or progesterone (1µM) or with both estradiol (10 nM) and progesterone (1µM) or left untreated as a control for 24 hours at 37°C in 5% CO_2 incubator [2]. After 24 hours, cells were washed with PBS and the total protein lysate was prepared using RIPA buffer (0.5M Tris-HCl, pH 7.4, 1.5M NaCl, 2.5% deoxycholic acid, 10% NP-40, 10mM EDTA) at 4°C.

Reference List

1. Chadchan,S.B., Kumar,V., Maurya,V.K., Soni,U.K., & Jha,R.K. Endoglin (CD105) coordinates the process of endometrial receptivity for embryo implantation. *Mol. Cell Endocrinol.* 425:69-83. doi: 10.1016/j.mce.2016.01.014. Epub;2016 Jan 21., 69-83 (2016).

2. Somkuti,S.G., Yuan,L., Fritz,M.A., & Lessey,B.A. Epidermal growth factor and sex steroids dynamically regulate a marker of endometrial receptivity in Ishikawa cells. *J. Clin. Endocrinol. Metab.* **82**, 2192-2197 (1997).
